# Supplementary material for: Auxin-Regulated Reversible Inhibition of TMK1 Signaling by MAKR2 Modulates the Dynamics of Root Gravitropism
Source: Curr Biol. 2021 Jan 11;31(1):228–237.e10. doi: 10.1016/j.cub.2020.10.011 (PMC7809621; doi:10.1016/j.cub.2020.10.011)
Supplement: Document S1. Figures S1–S5 [file mmc1.pdf]

Current Biology, Volume *31*

Supplemental Information

Auxin-Regulated Reversible Inhibition of TMK1 Signaling by MAKR2 Modulates the Dynamics of Root Gravitropism

Maria Mar Marquès-Bueno, Laia Armengot, Lise C. Noack, Joseph Bareille, Lesia Rodriguez, Matthieu Pierre Platre, Vincent Bayle, Mengying Liu, Davy Opdenacker, Steffen Vanneste, Barbara K. Möller, Zachary L. Nimchuk, Tom Beeckman, Ana I. Caño-Delgado, Jiří Friml, and Yvon Jaillais

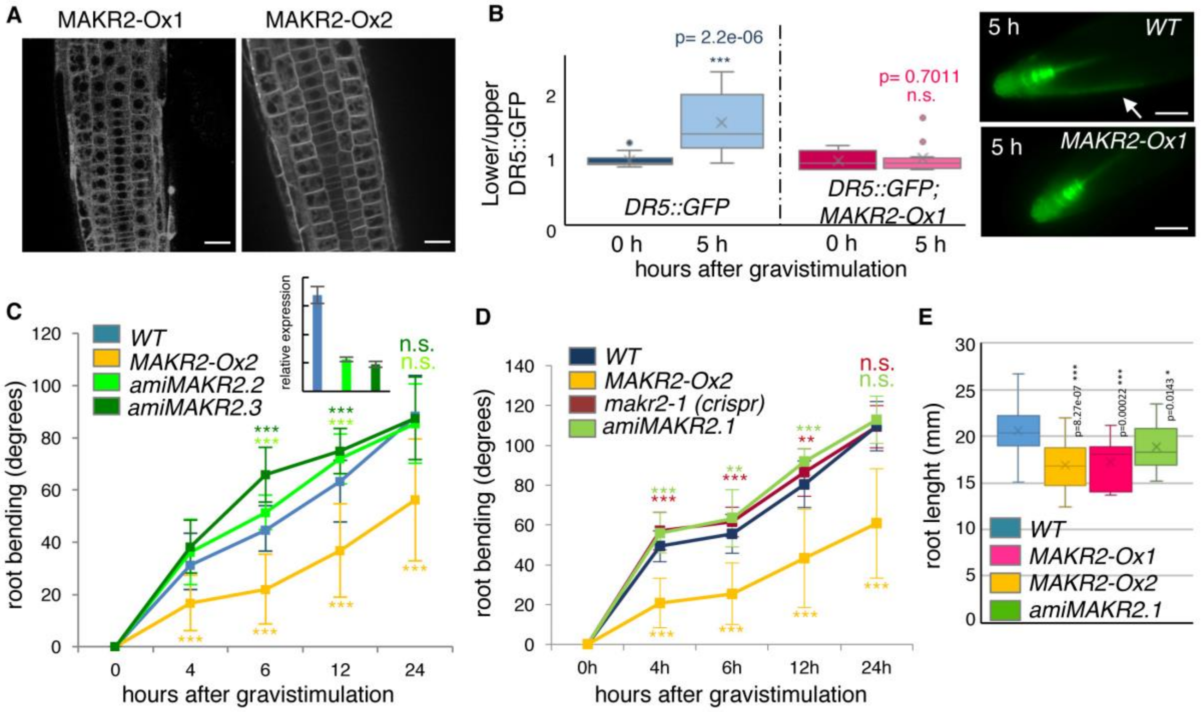

F

p-values related to Figure 1D

	6h	12h	24h	48h
MAKR2-Ox2	3.92e-07 ***	< 2e-16 ***	9.63e-10 ***	< 2e-16 ***
MAKR2-Ox1	9.93e-05 ***	7.38e-11 ***	0.00321 **	7.72e-09 ***
amiMAKR2.1	0.00184 **	0.00147 **	0.00848 **	0.983 n.s.

P-values related to Figure S1C

	4h	6h	12h	24h
MAKR2-Ox2	2.71e-10 ***	< 2.2e-16 ***	< 2.2e-16 ***	3.95E-15 ***
amiMAKR2.2	0.01531 *	2.03e-05 ***	1.69e-06 ***	0.7413 n.s.
amiMAKR2.3	0.0007991 ***	3.30e-05 ***	5.14e-11 ***	0.7967 n.s.

P-values related to Figure S1D

	4h	6h	12h	24h
MAKR2-Ox2	<2e-16 ***	< 2.2e-16 ***	< 2.2e-16 ***	<2.2e-16 ***
amiMAKR2.1	0.000245 ***	0.00124 **	4.57e-05 ***	0.157 n.s.
makr2-crispr	1.01e-05 ***	0.000374 ***	0.00794 **	0.902 n.s.

p-values related to Figure 2C

	6h	12h	24h
MAKR2-Ox1	1.37e-07 ***	6.42e-08 ***	2e-16 ***
ROP6-Ox	0.77 n.s.	0.00261 **	0.00379 **
MAKR2-Ox1;ROP6-Ox	0,119 n.s.	0,227 n.s.	0,0648 n.s.

Figure S1. Characterization and phenotyping of *MAKR2* gain- and loss-function mutants. Related to Figure 1 and 2. A) confocal picture showing the expression and localization of MAKR2-2Ch (*MAKR2-Ox1* line) and MAKR2-mCit (*MAKR2-Ox2* line). Scale bars: 30 µm. **B)** Quantification of DR5::GFP asymmetry (ratio of lower/upper) before and after 5 hours gravistimulation at a 135° angle and related representative fluorescent images after 5 hours of gravistimulation. The white arrow indicate the accumulation of the DR5rev::GFP signal on the lower part (gravistimulated side) of the root in the wild type. Scale bars: 50 µm. **C)** Kinetics of root gravitropic bending after reorienting seedlings of the genotypes indicated in the top left corner at a 135° angle. See S1F for a statistical comparison of each mutant genotype with the wild type at each time point. The inset represents qRT-PCR analyses of *MAKR2* expression in wild-type and two additional independent *amiMAKR2* lines (*amiMAKR2.2* light green and *amiMAKR2.3* dark green). **D)** Kinetics of root gravitropic bending after reorienting seedlings of the genotypes indicated in the top left corner at a 135° angle. See figure S1F for a statistical comparison of each mutant genotype with the wild type at each time point. **E)** Quantification of the primary root length of the indicated genotypes. All quantitative measurements presented in different graphs have been treated as independent experiments (e.g. different wild-type). **F)** Tables showing the p-value, corresponding to the statistical comparison of each mutant genotype with the wild type at each time point of the gravitropic kinetics presented in Figure 1D, Figure S1C, S1D and Figure 2C. Signif. codes: 'n.s.' p>0.05, '*' 0.05>p>0.01, '**' 0.01>p>0.001, '***' 0.001>p.

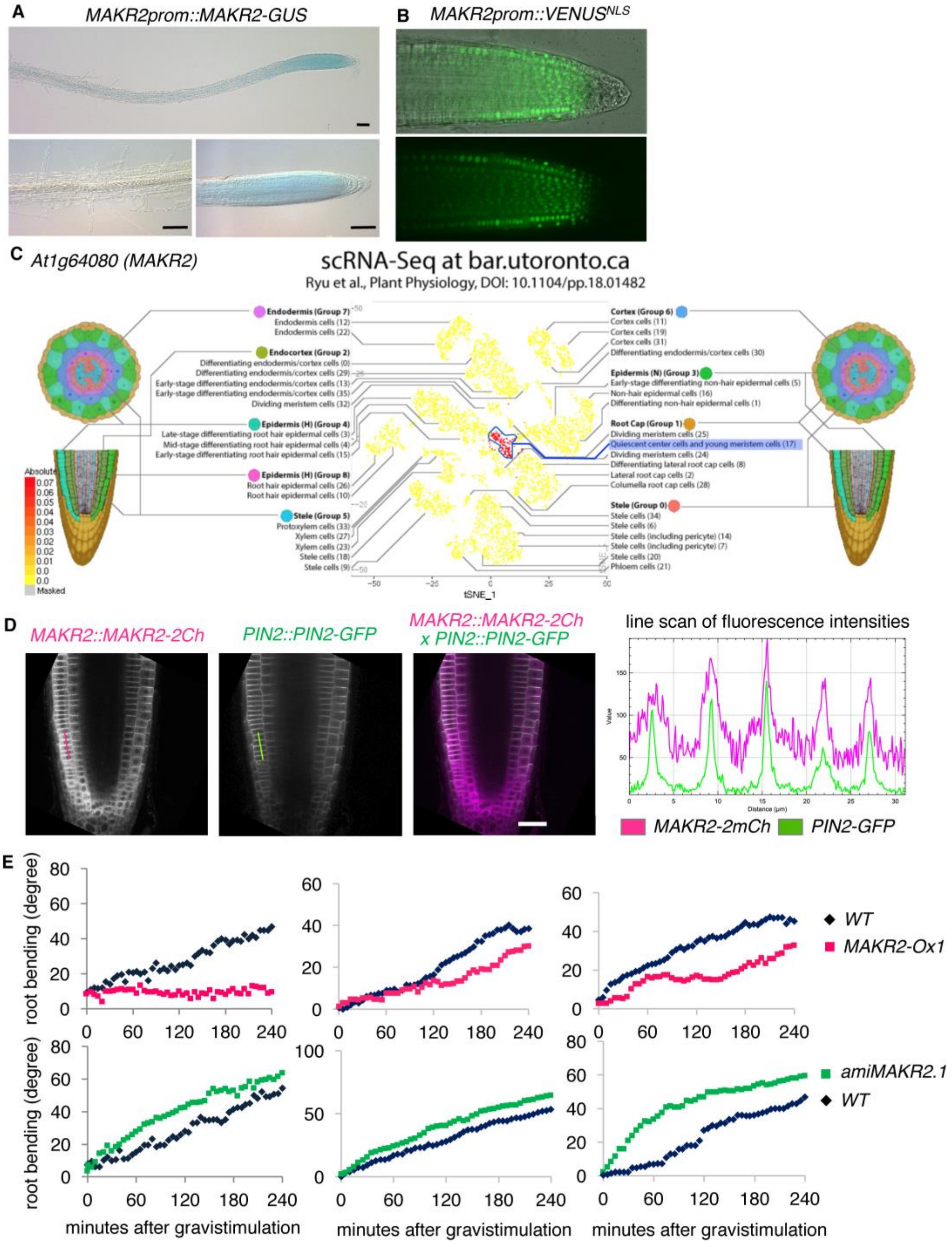

Figure S2. Analysis of *MAKR2* expression pattern in the root. Related to Figure 2. Microscopy pictures of roots showing the expression pattern of **(A)** *MAKR2prom::MAKR2-GUS*, scale bars: 100 µm and **(B)** *MAKR2prom::VENUS^{NLS}*, scale bar: 50 µm. **C)** Schematic representation of the results from single cell RNAseq for *MAKR2* expression in the root, showing that cells expressing *MAKR2* cluster together in a group of cells corresponding to quiescent center and young meristem cells. The graphic representation is from the Bio-Analytical Resource for Plant Biology (BAR, Toronto, https://bar.utoronto.ca/efp/cgi-bin/efpWeb.cgi?dataSource=Single_Cell) and the data are from [1]. **D)** Left: single channel (grey scale) of *MAKR2prom::MAKR2-2Ch* and *PIN2prom::PIN2-GFP* together with the corresponding overlay (MAKR2-2Ch in magenta, PIN2-GFP in green and colocalization in white). Right: line scan analysis of the fluorescent intensities in each channel. Note the overlapping pics, likely corresponding to the accumulation of both proteins at the plasma membrane. **E)** High resolution kinetics of the gravitropic response of *PIN2prom::PIN2-GFP*, *PIN2prom::PIN2-GFPxMAKR2-Ox1* and *PIN2prom::PIN2-GFP,xamiMAKR2.1* lines. Each graph shows the response of individual roots. See also Video S1 and S2.

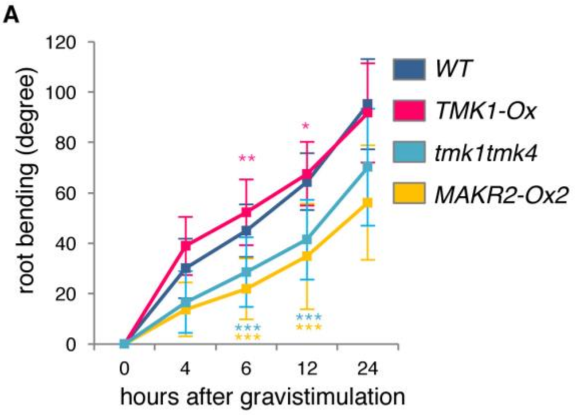

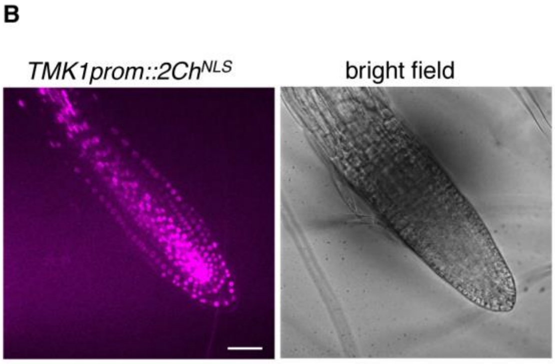

C

p-values related to Figure S3A

	6h	12h	24h
MAKR2-Ox2	1.03e-08 ***	5.82e-11 ***	1.93e-10 ***
TMK1ox	0.00824 **	0.0134 *	0,247
tmk1tmk4	3.26e-05 ***	5.05e-07 ***	8.51e-10 ***

p-values related to Figure 3A

	6h	12h	24h
MAKR2-Ox2	1.03e-08 ***	5.82e-11 ***	1.93e-10 ***
TMK1ox	0.00824 **	0.0134 *	0,247
TMK1ox;MAKR2-Ox2	0,729	0,556	0.0488 *

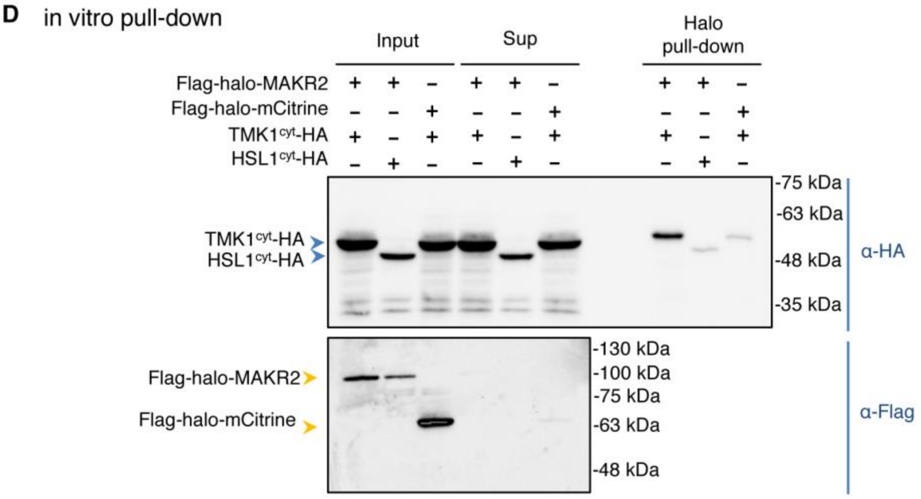

Figure S3. Quantitative analysis of the gravitropic phenotypes of *tmk1;tmk4* double mutant, expression pattern of *TMK1* and Halo-pull down with additional controls. Related to Figure 3. A) Kinetics of root gravitropic bending after reorienting seedlings of the genotypes indicated in the top left corner at a 135° angle. **B)** Confocal picture (left) and corresponding bright field image (right) showing the expression profile of *TMK1* in the *TMK1prom:2Ch*^{NLS} transcriptional reporter line. Scale bar: 50 µm. **C)** Table showing the p-value at each time point of the gravitropic kinetics presented in Figure S3A and Figure 3A. Data for WT, *MAKR2-Ox2* and *TMK1-Ox* are the same in graphs S3A and 3A. Signif. codes: 'n.s.' p>0.05, '*' 0.05>p>0.01, '**' 0.01>p>0.001, '***' 0.001>p. **D)** Pull-down assay using *in vitro* transcribed/translated proteins and Halo-tag purification. Co-purified proteins were visualized using an anti-HA antibody (labelled as Halo pull-down). The inputs (labelled Inputs) and supernatant (labelled Sup) were tested to show the relative amounts of Halo- and HA-tagged proteins and the binding efficiency to HaloLink magnetic beads (as described in [2]). TMK1^{cyt} and HSL1^{cyt} correspond to the isolated cytoplasmic domains and TMK1 and HSL1, respectively.

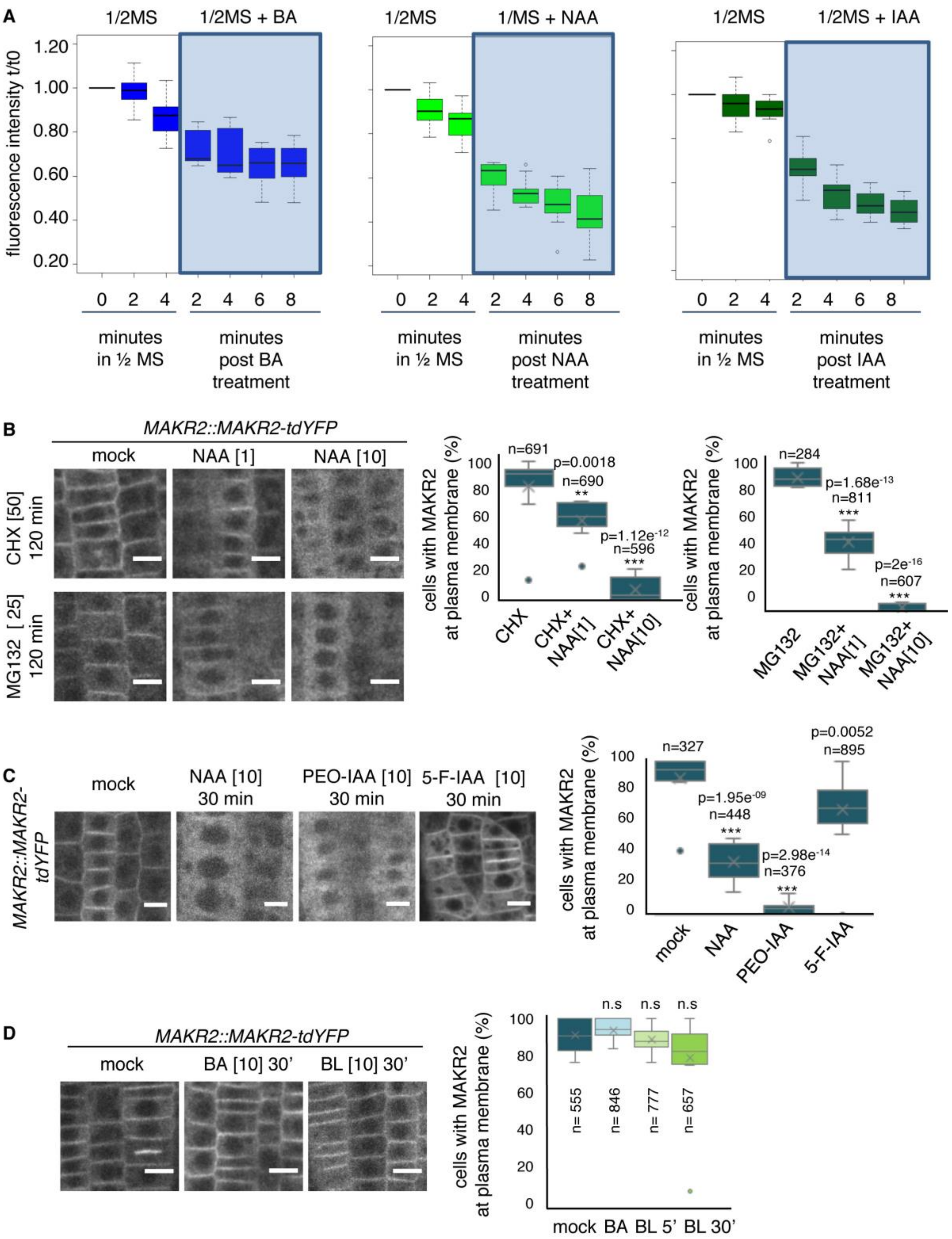

Figure S4. Time lapse analysis of MAKR2 membrane release upon auxin treatment and quantification of CHX, MG132, PEO-IAA, 5-F-IAA, BA, and BL effects on MAKR2-tdYFP localization. Related to Figure 4. A) Quantification of the MAKR2 localization at the plasma membrane and the cytosol after the application of NAA, IAA or benzoic acid (BA) treatment. Y-axis represents the ratio between the relative fluorescence at the plasma membrane at time t over the fluorescence at the plasma membrane at time t0 and the X-axis represents the time in minutes. The first four minutes shows the localization before the treatment and after either 10 µM NAA, 10 µM IAA or 10 µM benzoic acid was applied. Note that because we used a vertical confocal microscope with 20X objective with a low NA and a 488nm laser (rather than the high NA 40X objective and

515nm laser used in the rest of the experiments), we had to use a higher laser intensity accounting for increased background (i.e. fluorescence in intracellular bodies) and faster photobleaching. Comparison between the quantification of the NAA/IAA and the BA treatments allowed us to evaluate the extent of photobleaching vs auxin effect. See also Video S3. **B)** Confocal pictures and related quantifications of the *MAKR2prom::MAKR2-tdYFP* line pretreated for 120 min with the protein synthesis inhibitor cycloheximide (CHX at 50 µM) or the proteasome inhibitor MG132 (25 µM) and followed by a 30 min NAA/CHX or NAA/MG132 co-treatment at the indicated concentration of NAA. Scale bars: 10 µm. **C)** Confocal pictures of the *MAKR2prom::MAKR2-tdYFP* line following a 30 min treatment with EtOH (mock), NAA (10 µM), PEO-IAA (10 µM) and 5-F-IAA (10 µM) and related quantifications of the percentage of cells with MAKR2-tdYFP signal at the plasma membrane over the total number of cells. Scale bars: 10µm. **D)** Confocal pictures and related quantifications of *MAKR2prom::MAKR2-tdYFP* lines following a 30min treatment with either benzoic acid (BA) or brassinolide (BL). Scale bars: 10µm. n in each graph indicates the total number of cells counted.

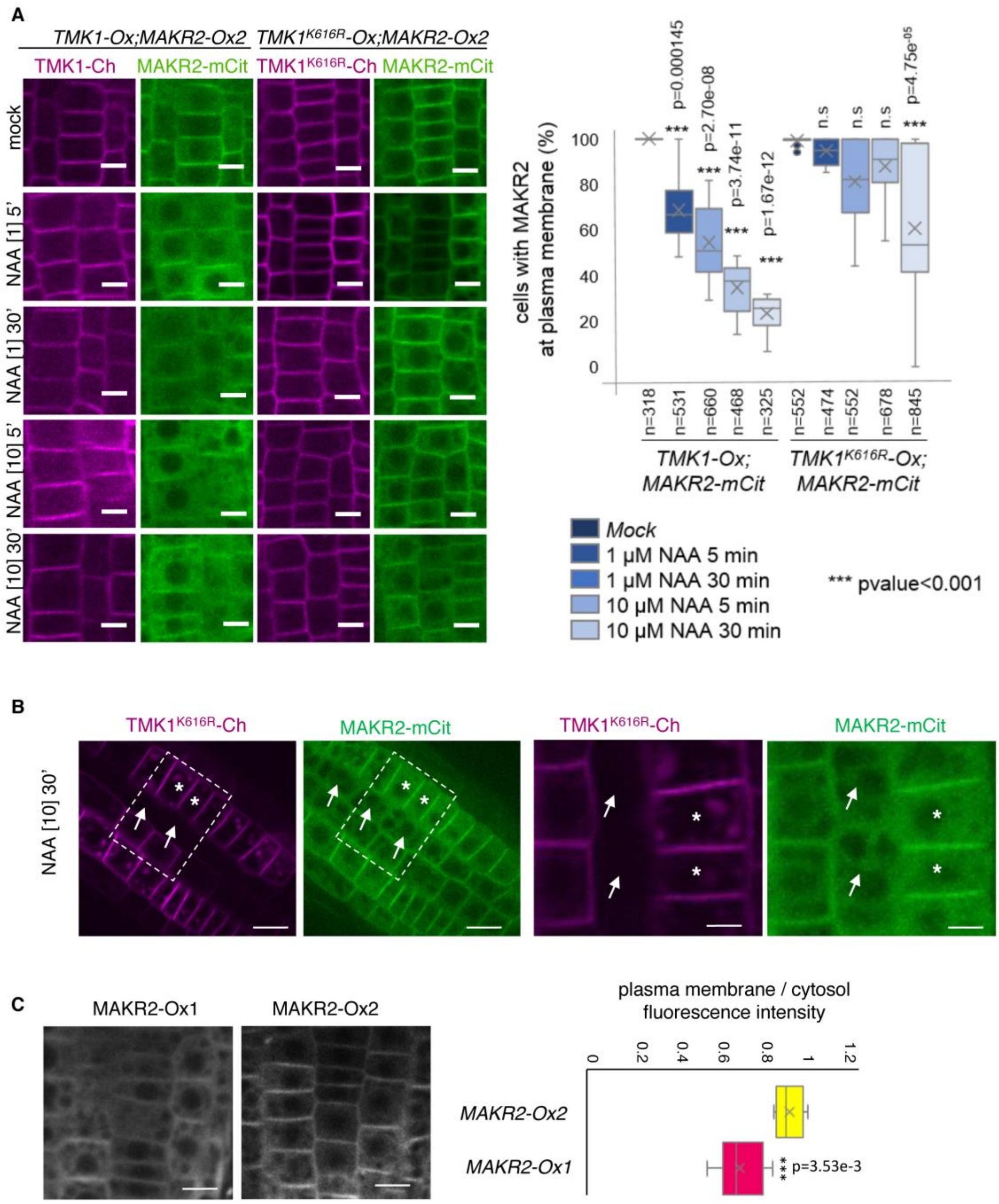

Figure S5. Quantification of TMK1-Ox and TMK1^{K616R}-Ox effect on MAKR2-mCit localization. related to Figure 4. A) Confocal pictures of roots coexpressing *UBQ10prom::TMK1-2xmCherry* (*TMK1-Ox*, left) and *2x35Sprom::MAKR2-mCitrine* (*MAKR2-Ox2*, right) (left) and *UBQ10prom::TMK1^{K616R}-2xmCherry* (*TMK1^{K616R}-Ox*, kinase dead, left) and *2x35Sprom::MAKR2-mCitrine* (*MAKR2-Ox2*, right) (right) according to the NAA treatment indicated on the left and related quantification. n indicates the number of cells counted. Note that the MAKR2-mCit picture for mock and NAA 10 µM 30 min are the same than in Figure 4C. Scale bars: 10 µm. **B)** Confocal pictures of roots coexpressing *2x35Sprom::MAKR2-mCitrine* (*MAKR2-mCit*) and *UBQ10prom::TMK1^{K616R}-2xmCherry* (*TMK1^{K616R}-Ox*, kinase dead) showing the mosaic expression of TMK1^{K616R}. The white asterisks indicate cells which express TMK1^{K616R}, and in which MAKR2-mCit does not relocalize to the cytosol following a 30-min treatment with 10 µM NAA. By contrast, the white arrows indicate cells which does not express TMK1^{K616R}, and in which MAKR2-mCit is cytosolic after a 30-min treatment with 10 µM NAA. Scale bars: 20 µm (left), 5 µm (right). **C)** Quantification of the fluorescence ratio of MAKR2 plasma membrane over cytosolic signal in *MAKR2-Ox1* and *MAKR2-Ox2* overexpression lines. Note that the relative amount of MAKR2 at the plasma membrane correlates with the level of expression (see Figure 1A) and with the respective phenotypes of these lines (see Figure 1B and 1D).

Supplemental Reference

S1. Ryu, K.H., Huang, L., Kang, H.M., and Schiefelbein, J. (2019). Single-Cell RNA Sequencing Resolves Molecular Relationships Among Individual Plant Cells. Plant physiology *179*, 1444-1456.

S2. Yazaki, J., Galli, M., Kim, A.Y., and Ecker, J.R. (2018). Profiling Interactome Networks with the HaloTag-NAPPA In Situ Protein Array. Curr Protoc Plant Biol *3*, e20071.
