# Supplementary material for: Auxin-Regulated Reversible Inhibition of TMK1 Signaling by MAKR2 Modulates the Dynamics of Root Gravitropism
Source: Curr Biol. 2021 Jan 11;31(1):228–237.e10. doi: 10.1016/j.cub.2020.10.011 (PMC7809621; doi:10.1016/j.cub.2020.10.011)
Supplement: Document S2. Article plus Supplemental Information [file mmc5.pdf]

Current Biology

Report

Auxin-Regulated Reversible Inhibition of TMK1 Signaling by MAKR2 Modulates the Dynamics of Root Gravitropism

Graphical Abstract

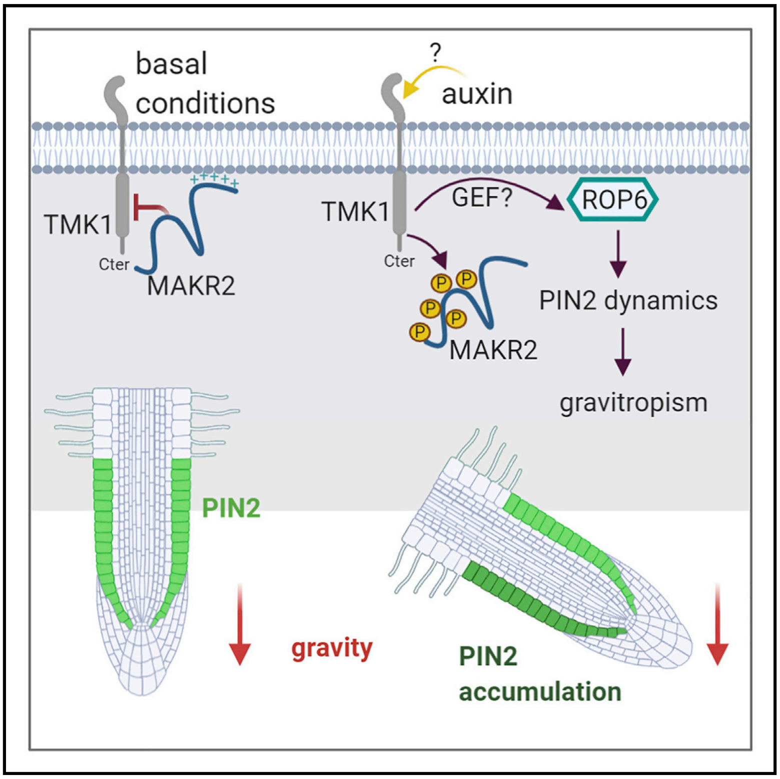

Highlights

- MAKR2 is co-expressed with PIN2 and regulates the pace of root gravitropism
- MAKR2 controls PIN2 asymmetric accumulation at the root level during gravitropism
- MAKR2 binds to and is a negative regulator of the TMK1 receptor kinase
- Auxin antagonizes the MAKR2 inhibition of TMK1 by delocalizing MAKR2 in the cytosol

Authors

Maria Mar Marquès-Bueno,
Laia Armengot, Lise C. Noack, ...,
Ana I. Caño-Delgado, Jiří Friml,
Yvon Jaillais

Correspondence

yvon.jaillais@ens-lyon.fr

In Brief

Marquès-Bueno, Armengot et al. show that the unstructured protein MAKR2 controls the dynamics of the root gravitropic response by acting as a negative regulator of the TMK1 receptor kinase. In addition, the MAKR2 inhibitory effect on TMK1 signaling is antagonized by auxin itself, which triggers rapid MAKR2 membrane dissociation in a TMK1-dependent manner.

Marquès-Bueno et al., 2021, Current Biology *31*, 228–237
January 11, 2021 © 2020 The Author(s). Published by Elsevier Inc.
https://doi.org/10.1016/j.cub.2020.10.011

CellPress

CellPress
OPEN ACCESS

Current Biology

Report

Auxin-Regulated Reversible Inhibition of TMK1 Signaling by MAKR2 Modulates the Dynamics of Root Gravitropism

Maria Mar Marquès-Bueno,[1,6,8] Laia Armengot,[1,8] Lise C. Noack,[1] Joseph Bareille,[1] Lesia Rodriguez,[2] Matthieu Pierre Platre,[1] Vincent Bayle,[1] Mengying Liu,[1] Davy Opdenacker,[3,4] Steffen Vanneste,[3,4,5] Barbara K. Möller,[3,4] Zachary L. Nimchuk,[7] Tom Beeckman,[3,4] Ana I. Caño-Delgado,[6] Jiří Friml,[2] and Yvon Jaillais[1,9,*]
[1]Laboratoire Reproduction et Développement des Plantes, Université de Lyon, ENS de Lyon, UCB Lyon 1, CNRS, INRAE, 69342 Lyon, France
[2]Institute of Science and Technology Austria (IST Austria), Am Campus 1, 3400 Klosterneuburg, Austria
[3]Center for Plant Systems Biology, VIB, Technologiepark 71, 9052 Ghent, Belgium
[4]Department of Plant Biotechnology and Bioinformatics, Ghent University, Technologiepark 71, 9052 Ghent, Belgium
[5]Lab of Plant Growth Analysis, Ghent University Global Campus, Songdomunhwa-Ro, 119, Yeonsu-gu, Incheon 21985, Republic of Korea
[6]Department of Molecular Genetics, Center for Research in Agricultural Genomics (CRAG), CSIC-IRTA-UAB-UB, Campus UAB, Bellaterra (Cerdanyola del Vallès), 08193 Barcelona, Spain
[7]Department of Biology, University of North Carolina at Chapel Hill, Chapel Hill, NC 27599, USA
[8]These authors contributed equally
[9]Lead Contact
*Correspondence: yvon.jaillais@ens-lyon.fr
https://doi.org/10.1016/j.cub.2020.10.011

SUMMARY

Plants are able to orient their growth according to gravity, which ultimately controls both shoot and root architecture.[1] Gravitropism is a dynamic process whereby gravistimulation induces the asymmetric distribution of the plant hormone auxin, leading to asymmetric growth, organ bending, and subsequent reset of auxin distribution back to the original pre-gravistimulation situation.[1–3] Differential auxin accumulation during the gravitropic response depends on the activity of polarly localized PIN-FORMED (PIN) auxin-efflux carriers.[1–4] In particular, the timing of this dynamic response is regulated by PIN2,[5,6] but the underlying molecular mechanisms are poorly understood. Here, we show that MEMBRANE ASSOCIATED KINASE REGULATOR2 (MAKR2) controls the pace of the root gravitropic response. We found that MAKR2 is required for the PIN2 asymmetry during gravitropism by acting as a negative regulator of the cell-surface signaling mediated by the receptor-like kinase TRANSMEMBRANE KINASE1 (TMK1).[2,7–10] Furthermore, we show that the MAKR2 inhibitory effect on TMK1 signaling is antagonized by auxin itself, which triggers rapid MAKR2 membrane dissociation in a TMK1-dependent manner. Our findings suggest that the timing of the root gravitropic response is orchestrated by the reversible inhibition of the TMK1 signaling pathway at the cell surface.

RESULTS AND DISCUSSION

Receptor-like kinases (RLKs) are involved in all aspects of plant life, including development, immunity, reproduction, and environmental interactions.[11] Yet we are still lacking mechanistic details on how those receptors are activated and regulated. Notably, the functions and mechanisms of RLK negative regulation have rarely been addressed, although it is established that receptor inhibition plays a critical role in signaling and diseases in metazoans.[12] BRI1 KINASE INHIBITOR1 (BKI1) is a plant-specific unstructured protein that negatively regulates the activity of the plant steroid receptor BRASSINOSTEROID INSENSITIVE1 (BRI1).[13–15] BKI1 acts through two evolutionarily conserved linear motifs: a C-terminal alpha helix of 20 residues that binds the BRI1 kinase domain and inhibits the interaction between BRI1 and its co-receptor BRI1 ASSOCIATED KINASE1 (BAK1),[13–16] and a cationic membrane hook, which targets BKI1 to the plasma membrane.[14,17] Upon brassinosteroid perception, BRI1 phosphorylates BKI1 on a conserved tyrosine within the membrane hook,[14] triggering an electrostatic switch that releases BKI1 from the plasma membrane into the cytosol[17,18] and allowing the transphosphorylation of BRI1/BAK1 kinases and subsequent activation of the pathway.[19]

The presence of both the cationic membrane hook and the BRI1-binding peptide at the C terminus defines a novel family of proteins named MEMBRANE ASSOCIATED KINASE REGULATOR (MAKR) composed of 7 members (BKI1 and MAKR1–MAKR6).[14] Like BKI1, these proteins are unstructured cytosolic proteins that are targeted to the plasma membrane via electrostatic interactions.[17] However, with the exception of MAKR1, they are unable to bind to BRI1 kinase and thus they likely control different signaling pathways.[14,20,21] For example, the auxin-inducible MAKR4 is involved in lateral root formation,[21] whereas MAKR5 is involved in protophloem differentiation.[20] The latter acts as a positive downstream effector of the leucine-rich-repeat (LRR) RLK BARELY ANY MERISTEM3 (BAM3), suggesting that

 © 2020 The Author(s). Published by Elsevier Inc.
This is an open access article under the CC BY license (http://creativecommons.org/licenses/by/4.0/).

MAKR proteins may act as positive or negative regulators of RLK signaling.[20,22]

Here, we addressed the function of MAKR2, a so far uncharacterized member of the MAKR family. We raised *MAKR2* gain- and loss-of-function transgenic *Arabidopsis* lines via overexpression and artificial microRNA (*amiMAKR2*), respectively. To monitor protein accumulation and localization, we fused MAKR2 at its C terminus with either the red fluorescent protein 2xmCHERRY (*2x35Sprom::MAKR2-2Ch*, hereafter designated as *MAKR2-Ox1*) or the yellow fluorescent protein mCITRINE (*2x35Sprom::MAKR2-mCit*, hereafter designated as *MAKR2-Ox2*). Quantitative RT-PCR and confocal analyses of tagged proteins confirmed the overexpression of *MAKR2* in *MAKR2-Ox* lines and its downregulation in each independent artificial microRNA line (Figures 1A, S1A, and S1C). The roots overexpressing *MAKR2* did not grow vertically, as manifested by an increased gravitropic index (Figure 1B). Analysis of the synthetic auxin output reporter *DR5prom::GUS* showed an arrow-like pattern of GUS accumulation at the root tip of *MAKR2-Ox1* lines with an increased signal on both flanks of the lateral root caps (Figure 1C). This DR5 arrow-like pattern at the root tip is typically observed in *pin2* loss-of-function alleles.[23,24] Consistent with a potentially impaired PIN-FORMED2 (PIN2) activity, *MAKR2* overexpression inhibited the establishment of the asymmetric patterns observed after 5 h of gravistimulation in both *DR5prom::GUS* and *DR5prom::GFP* reporter lines (Figures 1C and S1B). Quantitative analyses of root bending following gravistimulation showed that *MAKR2-Ox* lines reoriented slowly to the new gravity vector (Figure 1D). In contrast, three independent *amiMAKR2* lines had the opposite phenotype, displaying fast gravitropic bending (Figures 1D and S1C). To validate the specificity of our *amiMAKR2* lines, we created a crispr allele (*makr2-1*). This allele led to a truncated protein of 37 residues, comprising only the first 8 residues of MAKR2 and an additional 29 random residues. The *amiMAKR2* lines and the *makr2-1* allele had identical gravitropic phenotypes (Figure S1D). Both the *MAKR2-Ox* and *amiMAKR2.1* lines had slightly shorter primary roots than the wild type (Figure S1E); however, they had opposite gravitropic phenotypes (Figure 1D). Furthermore, we found no correlation between root length (Figure S1E) and the strength of the agravitropic phenotypes of the *MAKR2-Ox1* and *MAKR2-Ox2* lines (Figure 1D). Together, these data suggest that primary root growth is unlikely to explain the gravitropic phenotypes of *MAKR2* gain- and loss-of-function mutants.

The pace of root gravitropism is regulated by Rho GTPase of Plants 6 (ROP6), with the roots of *ROP6* loss-of-function mutants responding slowly to gravistimulation and the roots of *ROP6* gain-of-function mutants (e.g., *ROP6-Ox*) responding faster than wild-type roots.[8,9,25] Because the loss- and gain-of-function mutants of both *MAKR2* and *ROP6* have opposite phenotypes, we hypothesized that MAKR2 may act as a negative regulator of the ROP6 signaling pathway. To test this, we first assessed whether *MAKR2* is expressed in the same tissues as *ROP6* using a transcriptional reporter line, *MAKR2prom::VENUS^{NLS}*, and translational fusion lines, *MAKR2prom::MAKR2-tdYFP*, *MAKR2prom::MAKR2-GUS*, and *ROP6prom::mCitrine-ROP6*. *MAKR2* was expressed in the root meristem in the epidermis and cortex cell layers, as well as in the quiescent center and surrounding initials (Figures 2A and S2A–S2C). As reported with the GFP-ROP6 reporter lines,[8,26,27] we found that our mCit-ROP6 reporter was expressed in the root tip epidermis and cortex cells, albeit to a lower level than in internal tissues (Figure 2B). Therefore, *MAKR2* and *ROP6* expression partially overlap. Next, we addressed the genetic relationship between *ROP6* and *MAKR2* by crossing a *ROP6-Ox* line with our *MAKR2-Ox1* line, which show fast- and slow-gravitropic responses, respectively. *ROP6-Ox;MAKR2-Ox1* double transgenics showed a wild-type-like response, suggesting that *ROP6* overexpression mitigates the strong agravitropic phenotype induced by *MAKR2* overexpression (Figure 2C).

ROP6 has been proposed to mediate root gravitropic bending by regulating the trafficking of the auxin-efflux carrier PIN2.[2,4,8,9] In particular, ROP6 mediates PIN2 accumulation in the epidermis on the gravity-stimulated side of the root (lower side of the root, facing the new gravity vector).[2] We therefore analyzed whether MAKR2 could control PIN2 dynamics during gravitropism. First, we raised a *MAKR2prom::MAKR2-2Ch* line and crossed it with a *PIN2prom::PIN2-GFP* line to monitor their co-expression and localization. We found that MAKR2 and PIN2 were co-expressed and co-localized at the plasma membrane of root epidermis and cortex cells (Figure S2D). Next, we crossed the *MAKR2-Ox1* and *amiMAKR2.1* lines with the *PIN2prom::PIN2-GFP* reporter and followed PIN2-GFP accumulation during gravitropism using a vertical stereomicroscope setup.[28] High-resolution time-lapse analyses validated the slow- and fast-gravitropic response of *MAKR2-Ox1;PIN2prom::PIN2-GFP* and *amiMAKR2;PIN2-prom::PIN2-GFP*, respectively (Figure S2E; Videos S1 and S2). Using time-lapse analysis, we noticed that each root had slightly different gravitropic dynamics (Figure S2E), which made it difficult to pool the quantification of PIN2-GFP fluorescence between replicates. We thus decided to trace PIN2-GFP fluorescence in individual roots shown as independent replicates in Figure 2D. Importantly, although we observed some root-to-root variations in PIN2-GFP dynamics, the overall trend was nevertheless robust from replicate to replicate (Figure 2D). Quantitative measurements of PIN2-GFP fluorescence on the upper and lower sides of the root confirmed that PIN2-GFP is retained on the lower side of the root longer than on the upper side (Figure 2D; Videos S1 and S2).[2,29,30] In particular, PIN2-GFP signal at the lower part of the wild-type root initially increased before decreasing (Figure 2D). Such signal increase was not observed at the upper part of the root, which instead showed a steady decrease of fluorescence (Figure 2D). The differential PIN2-GFP accumulation between the upper and lower parts of the root was abolished in the *MAKR2-Ox1*-overexpressing line (Figure 2D; Video S1). By contrast, PIN2-GFP accumulation at the lower part of the root in the *amiMAKR2* line was more pronounced and lasted longer than in the wild type (Figure 2D; Video S2). Together, these results indicate that MAKR2, like ROP6, is required for dynamic PIN2 accumulation during root gravitropism, which could explain the relative gravitropic phenotypes of the *MAKR2-Ox* and *amiMAKR2* lines.

We next investigated whether MAKR2 may also regulate the activity of an RLK upstream of ROP6 activation. In the context of pavement-cell-shape morphogenesis, ROP6 acts downstream of the LRR RLKs from the TRANSMEMBRANE KINASE (TMK) family, which were proposed to operate as a relay for perception of extracellular auxin.[2,7,31] We thus wondered

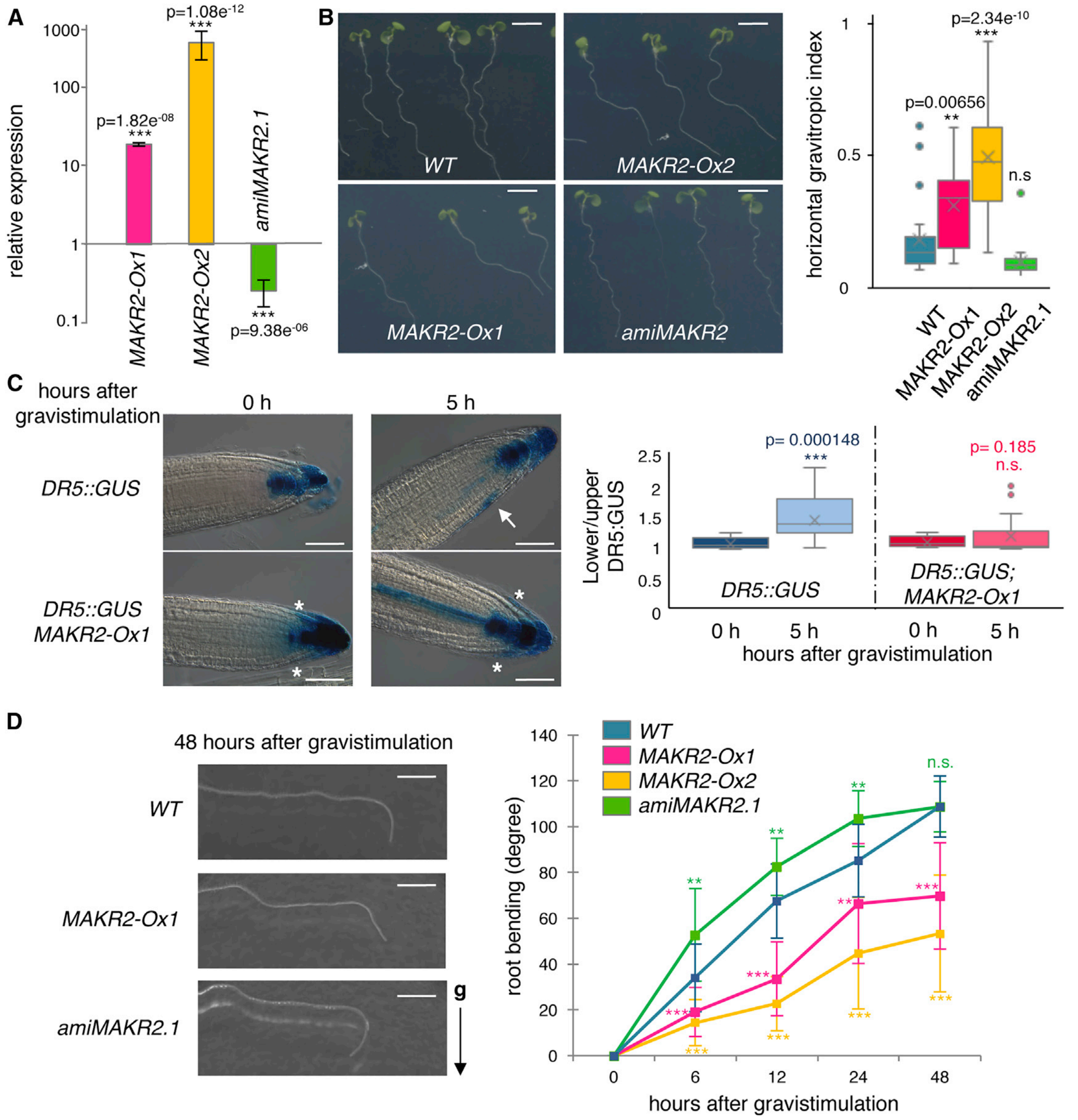

Figure 1. MAKR2 Regulates the Pace of the Root Gravitropic Response

(A) qRT-PCR analyses of *MAKR2* expression in *2x35Sprom::MAKR2-2xmCherry* (*MAKR2-Ox1*), *2x35Sprom::MAKR2-mCitrine* (*MAKR2-Ox2*), and *amiMAKR2.1* lines relative to *MAKR2* expression in wild-type seedlings (mean ± SEM).

(B) Pictures showing the root phenotypes of the genotypes indicated at the bottom and related quantification of the horizontal growth index (Tukey boxplot). Plants were grown at a 45° angle with respect to the vertical axis. Scale bars represent 5 mm.

(C) *DR5prom::GUS* accumulation pattern in the absence and after 5 h of gravistimulation at a 135° angle in wild-type and *MAKR2-Ox1* plants and related quantification (Tukey boxplot). The white asterisks indicate the arrow-like pattern observed in *MAKR2-Ox1* lines; the white arrow indicates the asymmetric GUS signal observed after gravistimulation in the wild type. Scale bars represent 50 μm.

(D) Representative pictures of the root gravitropic curvature 48 h after reorienting seedlings at a 135° angle and related quantification of root gravitropic bending over time (mean ± SEM). Scale bars represent 2 mm. See Figure S1F for a statistical comparison.

For the horizontal gravitropic index and the kinetics of the gravitropic response, a linear model was fitted on measurements from wild-type plants and the different mutants using lm() function from stats package available in R software (https://www.r-project.org/). This model estimates a weight for each variable (wild-type and mutant plants) and the associated probability that such weight is different from zero based on a t test. The probability derived from the t test is the p value in this comparison and significant differences were considered when $p < 0.05$. See also Figure S1.

whether TMKs may also participate in root gravitropism. TMKs form a family of redundant receptors, with single mutants having no or subtle root phenotypes,[32] whereas higher-order mutants have strong pleiotropic developmental defects.[33] The *tmk1;tmk4* double mutant showed a reduced root gravitropic response (Figure S3A). Although consistent with the notion that TMK receptors may be involved in root gravitropic bending, the *tmk1;tmk4* mutant also had stunted root growth,[33] making it difficult

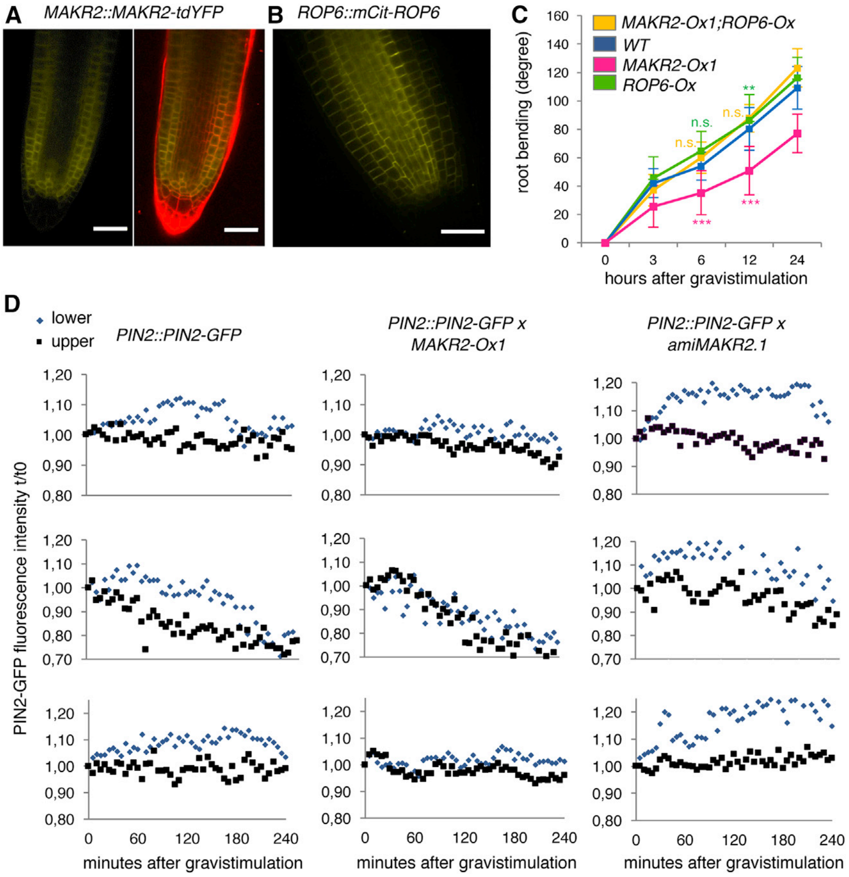

Figure 2. MAKR2 Mediates PIN2-GFP Dynamic Accumulation during Gravitropism

(A) Confocal pictures of the *MAKR2prom::MAKR2-tdYFP* line showing the MAKR2-tdYFP localization and expression pattern at the root tip. Left: yellow fluorescent protein (YFP) channel; right: overlay between YFP channel (yellow) and membranes counterstained by FM4-64 (red).

(B) Confocal pictures of the complemented *ROP6prom::mCitrine-ROP6/rop6-2* line showing the mCit-ROP6 localization and expression pattern at the root tip.

(C) Kinetics of root gravitropic bending after reorienting seedlings at a 135° angle. See Figure S1F for a statistical comparison. A linear model was fitted on measurements from wild-type plants and the different mutants using lm() function from stats package available in R software (https://www.r-project.org/). This model estimates a weight for each variable (wild-type and mutant plants) and the associated probability that such weight is different from zero based on a t test. The probability derived from the t test is the p value in this comparison and significant differences were considered when $p < 0.05$.

(D) Quantification of PIN2-GFP in the upper (blue diamonds) and lower (black squares) part of the root in the *PIN2prom::PIN2-GFP*, *PIN2prom::PIN2-GFP;MAKR2-Ox1*, and *PIN2prom::PIN2-GFP;amiMAKR2.1* lines. Each graph shows the response in a single individual root (see also Videos S1 and S2). In each case, fluorescence intensities were normalized with respect to the initial fluorescence value (time 0 min).

Scale bars represent 30 μm.

See also Figures S1 and S2 and Videos S1 and S2.

to conclude whether its gravitropic phenotype was a primary or secondary phenotype. A transcriptional reporter line, *TMK1prom::2Ch^{NLS}*, confirmed that *TMK1* is expressed in all the tissues of the root meristem, including the epidermis and cortex, where *MAKR2*, *PIN2*, and *ROP6* are also expressed (Figure S3B). We therefore analyzed the phenotype of *TMK1* overexpression by generating a TMK1-2xmCherry line driven under the control of the ubiquitous promoter of the *UBIQUITIN10* gene (*UBQ10prom::TMK1-2Ch*, hereafter referred to as *TMK1-Ox*). Similar to the *ROP6-Ox* and *amiMAKR2* lines, the *TMK1-Ox* line displayed a fast root gravitropic response (Figure 3A). Together, the *tmk1;tmk4* loss-of-function and *TMK1-Ox* gain-of-function phenotypes suggest that TMK1 may act upstream of ROP6 signaling both for pavement cell morphogenesis in leaves and in root gravitropism. Interestingly, *TMK1-Ox; MAKR2-Ox2* double transgenics had a wild-type-like root gravitropic response (Figure 3A). This genetic analysis shows that overexpression of *MAKR2* is able to counteract the phenotypic effects of *TMK1* overexpression, suggesting that MAKR2 may act as a negative regulator of TMK1, upstream of ROP6 activation.

We next tested whether MAKR2 interacts with TMK1. We found that Flag-Halo-MAKR2 directly interacted with the isolated cytosolic domain of TMK1 *in vitro* (TMK1^{cyt}-HA; hemagglutinin), whereas Flag-Halo-mCitrine did not (Figure 3B). This interaction was specific for the cytosolic domain of TMK1, as the cytosolic domain of HAESA-LIKE1 (HSL1^{cyt}-HA), which has a related kinase domain to TMK1, did not interact with Flag-Halo-MAKR2 (Figure S3D). Furthermore, full-length TMK1 (TMK1-3HA), but not a kinase-deleted version (TMK1$^{\Delta kinase}$-3HA), co-immunoprecipitated with MAKR2-mCit when co-expressed in protoplasts (Figure 3C). One hour of auxin treatment (1 μM indole 3-acetic acid; IAA) did not have a strong effect on the interaction between TMK1-3HA and MAKR2-mCit in this protoplast assay (Figure 3C). Next, we tested whether MAKR2 interaction with TMK1 was dependent upon its kinase activity and whether TMK1 may phosphorylate MAKR2. To this end, we co-expressed in bacteria HA-MAKR2 with the isolated cytosolic domain of TMK1 in its wild-type or kinase-dead form (6His-Flag-TMK1^{cyt} and 6His-Flag-TMK1$^{cyt-K616R}$, respectively). We purified both TMK1 proteins on a nickel column and identified potential interacting proteins by mass spectrometry. We found MAKR2 to be the only protein represented by more than one peptide to co-purify with both active and inactive TMK1 kinase domains. In each case, we recovered 13 unique peptides in MAKR2, representing a 42% peptide coverage (63% coverage for both TMK1^{cyt} and TMK1$^{cyt-K616R}$) (Figure 3D). These results indicate that MAKR2 directly interacts with the kinase domain of TMK1 *in vitro*, irrespective of its kinase activity. We next assessed the phosphorylation status of each peptide recovered during the mass spectrometry experiments. Such analysis of the phosphosites revealed that the MAKR2 C terminus was phosphorylated

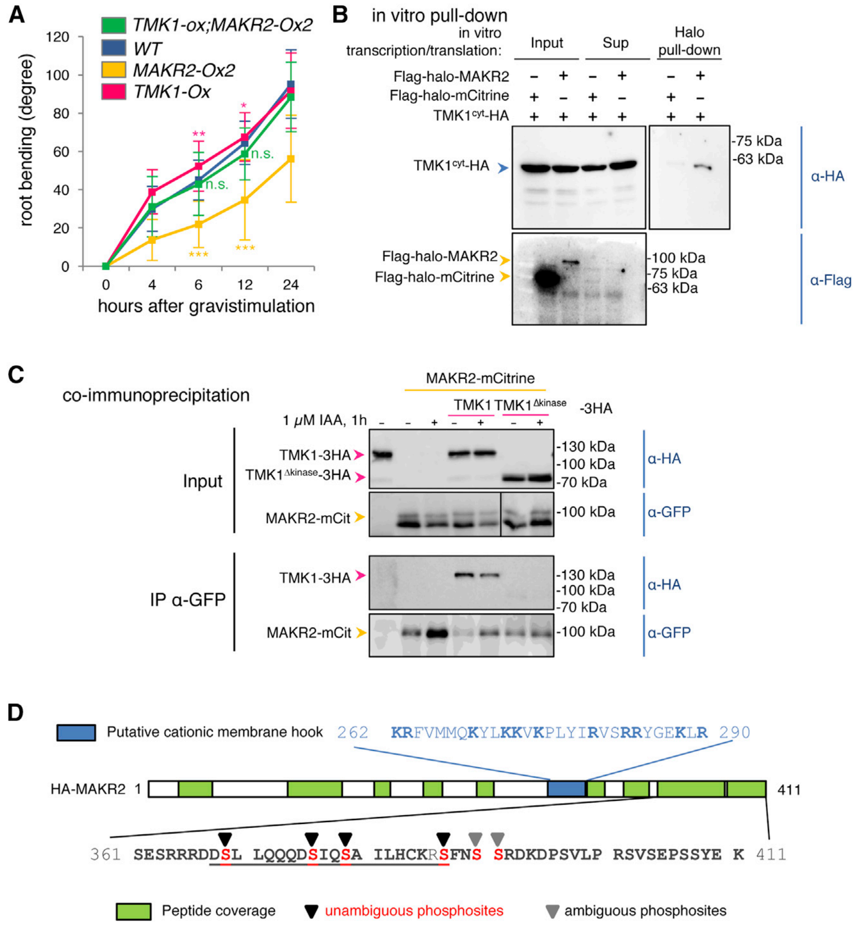

Figure 3. TMK1 Interacts with and Phosphorylates MAKR2 and Acts Upstream of MAKR2 in the Regulation of Root Gravitropism

(A) Kinetics of root gravitropic bending after reorienting seedlings of the genotypes indicated in the top left corner at a 135° angle. See Figure S3C for a statistical comparison. A linear model was fitted on measurements from wild-type plants and the different mutants using lm() function from stats package available in R software (https://www.r-project.org/). This model estimates a weight for each variable (wild-type and mutant plants) and the associated probability that such weight is different from zero based on a t test. The probability derived from the t test is the p value in this comparison and significant differences were considered when $p < 0.05$.

(B) Pull-down assay using *in-vitro*-transcribed/translated proteins and Halo-tag purification. Co-purified proteins were visualized using an anti-HA antibody (labeled Halo pull-down). The inputs (labeled Inputs) and supernatant (labeled Sup) were tested to show the relative amounts of Halo- and HA-tagged proteins and the binding efficiency to HaloLink magnetic beads (as described in Yazaki et al.[34]). TMK1^{cyt} corresponds to the isolated TMK1 cytoplasmic domain.

(C) Co-immunoprecipitation of full-length TMK1-3HA but not TMK1$^{\Delta kinase}$-3HA with MAKR2-mCitrine. Immunoprecipitation (IP) of MAKR2-mCitrine with an anti-GFP antibody and immunoblotting (IB) using an anti-GFP antibody or anti-HA antibody. Protoplasts were incubated or not for 1 h with 1 μM IAA.

(D) The scheme represents the MAKR2 protein, with the peptides recovered by mass spectrometry highlighted in green and the phosphorylation sites shown by arrowheads (only found with active TMK1^{cyt} but not inactive TMK1$^{cyt\text{-}K616R}$). Black arrowheads indicate phosphorylation sites that could be determined with 100% accuracy, whereas gray arrowheads indicate ambiguity on which of the two consecutive serines is phosphorylated. The residues corresponding to the conserved C-terminal tail are underlined. The blue box indicates the position of the putative cationic membrane hook, and the corresponding Arg/Lys residues are highlighted in bold.

See also Figure S3.

on 5 serines when it was co-expressed and co-purified with TMK1^{cyt} (Figure 3D). By contrast, we found no phosphorylation sites when MAKR2 was co-expressed and co-purified with TMK1$^{cyt\text{-}K616R}$. Altogether, our results suggest that MAKR2 directly interacts with and is phosphorylated by TMK1 *in vitro*, and interacts with full-length TMK1 *in vivo*.

MAKR2prom::MAKR2-tdYFP expression was too low to follow its localization during the gravitropic response, which provides an endogenous auxin treatment on the lower (i.e., gravistimulated) side of the root. As an alternative, we followed MAKR2 localization after exogenous auxin treatments. We found a time- and dose-dependent effect of the synthetic auxin 1-naphthaleneacetic acid (NAA), which induced the relocalization of *MAKR2prom::MAKR2-tdYFP* into the cytosol as early as 5 min post treatment (Figure 4A). This rapid effect of auxin treatment was also observed after 5 min of IAA application (Figure 4A). In addition, microfluidics, coupled with time-lapse imaging of *MAKR2prom::MAKR2-tdYFP* localization, confirmed that this effect was rapid (i.e., within 2 min following either NAA or IAA treatment) (Figures 4B and S4A; Video S3).[35] MAKR2 released into the cytosol was independent of protein translation, as shown by pretreatment with the protein synthesis inhibitor cycloheximide (CHX) (Figure S4B). Furthermore, inhibition of protein degradation by the proteasome inhibitor MG-132, which is required for auxin-mediated gene expression regulation by the TIR1 family,[36] had no effect on auxin-triggered MAKR2 plasma membrane dissociation (Figure S4B). This suggested that the MAKR2 plasma-membrane-to-cytosol localization switch was regulated by a potential "non-transcriptional" arm of auxin signaling at or close to the plasma membrane, rather than by a TIR1-mediated regulation of transcription.[2] Accordingly, the auxin antagonist PEO-IAA, known to inhibit TIR1-mediated nuclear activity but not the non-transcriptional arm of auxin signaling,[29,37] efficiently displaced MAKR2 away from the plasma membrane (Figure S4C). By contrast, 5-F-IAA, an auxin analog that is able to activate TIR1-mediated gene expression but not the ROP6 pathway,[29,37] had little effect on MAKR2 localization (Figure S4C). Furthermore, benzoic acid (BA), an inactive auxin analog with a pKa similar to that of NAA, and brassinolide had no effect on *MAKR2prom::MAKR2-tdYFP* localization (Figure S4D). To address whether this auxin effect was dependent upon the TMK receptors and to bypass the problem due to

CellPress
OPEN ACCESS

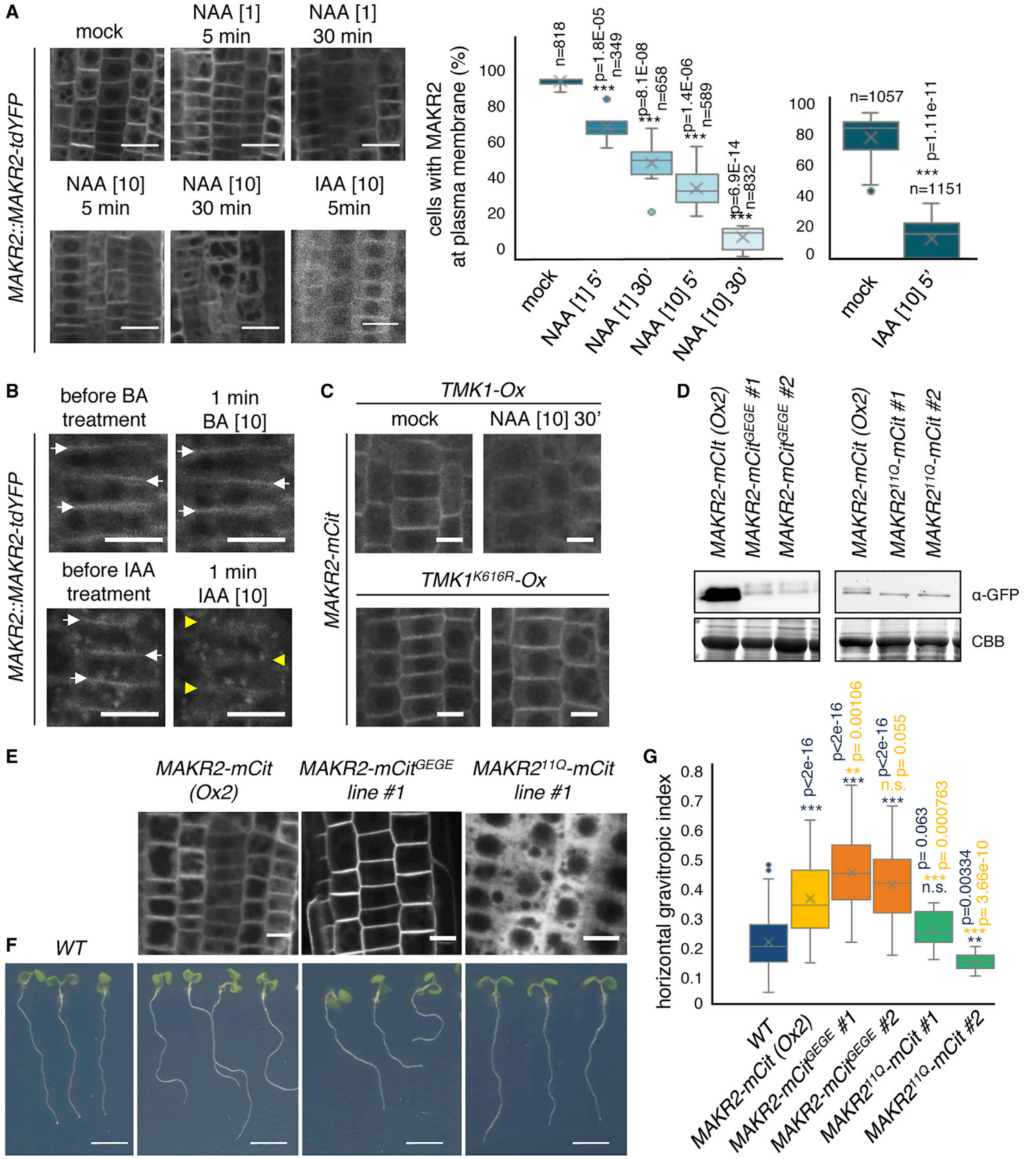

Figure 4. Auxin Triggers MAKR2 Plasma Membrane Dissociation in a TMK1-Dependent Manner to Antagonize MAKR2 Inhibitory Activity

(A) Confocal pictures of the *MAKR2prom::MAKR2-tdYFP* line following NAA or IAA treatment for the time and concentration indicated in each panel and related quantification. n indicates the number of cells counted. A pairwise comparison between mock plants and plants subjected to different treatments was performed using a t test with Welch's correction to account for unequal variances using R software (https://www.r-project.org/). The probability derived from the t test is the p value in this comparison and significant differences were considered when $p < 0.01$.

(B) Successive confocal pictures of the *MAKR2prom::MAKR2-tdYFP* line before and after 1 min of benzoic acid (BA; control) or IAA treatment (Video S3). White arrows indicate MAKR2 plasma membrane localization, whereas the yellow arrowheads show MAKR2 disappearance from the plasma membrane upon IAA but not BA treatment.

(legend continued on next page)

functional redundancy between the four TMK family members, we used a dominant-negative strategy, by overexpressing a kinase-dead version of full-length TMK1 (TMK1^{K616R}).[7,38] TMK1^{K616R} overexpression severely impaired the auxin-induced MAKR2-mCit release from the plasma membrane, whereas overexpression of the wild-type version of TMK1 did not (Figures 4C and S5A). Note that *TMK1^{K616R}-mCH*-overexpressing plants had a mosaic expression and that the inhibition of MAKR2-mCit released from the plasma membrane into the cytosol following auxin treatment was only observed in cells expressing TMK1^{K616R}-mCh (Figure S5B). Together, these results suggest that auxin rapidly regulates MAKR2 localization, via a non-transcriptional, TMK-dependent mechanism.

We next addressed the functional impact of the MAKR2 localization switch on root gravitropism. To this end, we first quantified the relative plasma membrane and cytosolic localization of MAKR2 in the *MAKR2-Ox1* and *MAKR2-Ox2* lines (Figure S5C). We found that MAKR2 in *MAKR2-Ox1*, which is less expressed than in the *MAKR2-Ox2* line (Figure 1A), had a more pronounced localization in the cytosol and accumulated less at the plasma membrane (Figure S5C). This result is consistent with a model in which MAKR2 inhibits the activity of TMK1, which itself triggers the relocalization of MAKR2 in the cytosol. Indeed, according to this model, a strong *MAKR2* overexpression would lead to a strong inhibition of TMK1 activity, which would not be able to induce the efficient release of MAKR2 into the cytosol. By contrast, a mild overexpression of *MAKR2* would lead to a milder inhibition of TMK1, which would be able to trigger more MAKR2 release from the plasma membrane into the cytosol. To further test this model, we engineered MAKR2 mutant versions that are constitutively localized at the plasma membrane or in the cytoplasm. To lock MAKR2 at the plasma membrane, we added a C-terminal geranylgeranylation sequence (MAKR2-mCit^{GEGE}). MAKR2-mCit^{GEGE} was exclusively found at the plasma membrane, by comparison with MAKR2-mCit, which localized both to the plasma membrane and in the cytosol (Figure 4E). Lines overexpressing *MAKR2-mCit^{GEGE}* had a strong agravitropic phenotype, similar to or even stronger than the *MAKR2-Ox2* lines (Figures 4F and 4G), whereas their expression levels were much lower (Figure 4D). This result indicates that a MAKR2 protein constitutively localized at the plasma membrane is extremely potent to inhibit root gravitropism. We previously showed that MAKR2 interacts with anionic lipids *in vitro* and relies on plasma membrane electrostatics for plasma membrane localization in yeast and *Arabidopsis*.[17] To inhibit MAKR2 membrane-binding ability, we mutated the 11 lysine and arginine residues within its putative membrane hook (Figure 3D) into neutral glutamines (MAKR2^{11Q}-mCit). MAKR2^{11Q}-mCit was cytosolic (Figure 4E), confirming that MAKR2 likely localizes to the plasma membrane through electrostatic interactions with anionic lipids. Importantly, unlike *MAKR2-Ox2*, *MAKR2^{11Q}-mCit*-overexpressing lines did not have an elevated horizontal gravitropic index even when expressed at similar levels (Figures 4D–4G). Together, these results suggest that MAKR2 inhibits gravitropism at the plasma membrane, likely through its interaction with the TMK1 receptor, and that auxin, via the activity of the TMK1 receptor itself, antagonizes this inhibition by triggering the relocalization of MAKR2 into the cytosol.

Altogether, our results suggest that MAKR2 acts as an upstream negative regulator of ROP6 during the gravitropic response by directly interacting with the TMK1 kinase domain. However, the exact mechanism by which TMK1/MAKR2 regulate ROP6 activity remains unresolved. Both TMK1 and ROP6 have been shown to be involved in fast non-transcriptional auxin response,[7–9,25,32] although the exact mode of auxin perception at or near the cell surface is still unclear.[2] We propose that the MAKR2-mediated negative regulation of TMK1 activity is counterbalanced by auxin itself, which triggers MAKR2 relocalization from the plasma membrane into the cytosol. It is possible that this relocalization is induced by TMK1-mediated phosphorylation. However, it is unlikely that the phosphorylation sites that we uncovered in the MAKR2 C-terminal tail are directly involved in the regulation of MAKR2 localization, because they are far away from the MAKR2 membrane hook and their exact function remains to be experimentally determined.

Although BKI1 and MAKR5 appear to essentially work as inhibitor and activator, respectively, of the BRI1 and BAM3 signaling pathways,[15,20] our results suggest a parallel mode of action of MAKR2 and BKI1 in downregulating TMK1 and BRI1 signaling at the cell surface, respectively. We propose that MAKR2/TMK antagonist activity allows finely tuning ROP6 signaling during the gravitropic response and thereby regulates the timing of root bending in response to gravity. Together, our results emphasize the importance of RLK negative regulation, which appears critical to determine the strength and dynamics of the output signal.

STAR★METHODS

Detailed methods are provided in the online version of this paper and include the following:

(C) Confocal pictures showing MAKR2-mCit localization (*MAKR2-Ox2*) in *UBQ10prom::TMK1-2xmCherry* (*TMK1-Ox*) and *UBQ10prom::TMK1^{K616R}-2xmCherry* (*TMK1^{K616R}-Ox*, kinase dead) in the absence or presence of NAA at the indicated time and concentration.

(D) Anti-GFP western blots showing the relative accumulation of MAKR2-mCit (in the *MAKR2-Ox2* line) and two independent transgenic lines overexpressing MAKR2-mCit^{GEGE} and MAKR2^{11Q}-mCit. CBB, Coomassie brilliant blue.

(E) Confocal pictures comparing the localization of *2x35Sprom::MAKR2-mCit* (*MAKR2-Ox2*), *2x35Sprom::MAKR2-mCit^{GEGE}* (MAKR2-mCit^{GEGE}, constitutively tethered to the plasma membrane), and *2x35Sprom::MAKR2^{11Q}-mCit* (MAKR2^{11Q}-mCit, constitutively cytoplasmic).

(F and G) Pictures showing the root phenotypes of the genotypes indicated at the top (F) and related quantification of the horizontal gravitropic index (G). Statistical comparison with the wild type (WT) is in blue and with *MAKR2-Ox2* is in yellow. In (G), a linear model was fitted on measurements from wild-type plants and the different mutants using lm() function from stats package available in R software (https://www.r-project.org/). This model estimates a weight for each variable (wild-type and mutant plants) and the associated probability that such weight is different from zero based on a t test. The probability derived from the t test is the p value in this comparison and significant differences were considered when $p < 0.05$.

Scale bars represent 20 μm (A), 10 μm (B, C, and E), and 5 mm (F).

See also Figures S4 and S5 and Video S3.

CellPress
OPEN ACCESS

- KEY RESOURCES TABLE
- RESOURCE AVAILABILITY
  - Lead Contact
  - Materials Availability
  - Data and Code Availability
- EXPERIMENTAL MODEL AND SUBJECT DETAILS
  - Plant material and Growth conditions
  - Plant Transformation and Selection
- METHOD DETAILS
  - Cloning and characterization of transgenic lines
  - > amiMAKR2
  - Gravitropism experiments
  - Quantitative RT-PCR (qPCR)
  - Drugs and hormones treatments
  - Microscopy
  - *In vitro* Halo-pull down
  - Co-expression, purification and mass-spectrometry
  - Co-immunoprecipitation Assay
  - Image Quantification
- QUANTIFICATION AND STATISTICAL ANALYSIS
  - Phenotyping
  - Cell biology experiments

SUPPLEMENTAL INFORMATION

Supplemental Information can be found online at https://doi.org/10.1016/j.cub.2020.10.011.

ACKNOWLEDGMENTS

We thank the SiCE group for discussions and comments; S. Yalovsky, B. Scheres, and the NASC/ABRC collection for providing transgenic *Arabidopsis* lines and plasmids; L. Kalmbach and M. Barberon for the gift of *pLOK180_pFR7m34GW*; A. Lacroix, J. Berger, and P. Bolland for plant care; and M. Fendrych for help with microfluidics in the J.F. lab. We acknowledge the contribution of the SFR Biosciences (UMS3444/CNRS, US8/Inser m, ENS de Lyon, UCBL) facilities: C. Lionet, E. Chatre, and J. Brocard at LBI-PLATIM-MICROSCOPY for assistance with imaging, and V. Guegen-Chaignon and A. Page at the Protein Science Facility (PSF) for assistance with protein purification and mass spectrometry. Y.J. was funded by ERC grant 3363360-APPL under FP/2007–2013. Y.J. and Z.L.N. were funded by an ANR- and NSF-supported ERA-CAPS project (SICOPID: ANR-17-CAPS-0003-01/NSF PGRP IOS-1841917). A.I.C.-D. is funded by an ERC consolidator grant (ERC-2015-CoG–683163) and BIO2016-78955 grant from the Spanish Ministry of Economy and Competitiveness. Exchanges between the Y.J. and T.B. laboratories were funded by Tournesol grant 35656NB. B.K.M. was funded by the Omics@vib Marie Curie COFUND and Research Foundation Flanders for a postdoctoral fellowship.

AUTHOR CONTRIBUTIONS

M.M.M.-B., L.A., and M.L. performed cloning and raised and characterized the genetic materials; M.M.M.-B., L.A., and L.C.N. quantified gravitropic bending and performed western blot analyses of protein accumulation in MAKR2 mutant lines; M.M.M.-B. performed all microscopy experiments (double-blind quantifications in collaboration with L.A.); M.M.M.-B. performed DR5::GUS and DR5::GFP analyses in A.I.C.-D.'s laboratory; M.M.M.-B. performed the kinematic analysis of PIN2-GFP during gravitropism in T.B.'s laboratory (with the help of D.O., B.K.M., and S.V.); M.M.M.-B. performed the microfluidics experiments in J.F.'s laboratory; J.B. and L.A. performed *in vitro* Halo-pull down and co-expression/purification in bacteria and related cloning; L.A. designed, isolated, characterized, and phenotyped the *makr2-1* crispr allele with the assistance of Z.L.N.; L.R. performed co-immunoprecipitation in J.F.'s laboratory; M.P.P. provided genetic materials; V.B. assisted with microscopy and image quantification; Y.J. supervised research; M.M.M.-B., L.A., and Y.J. conceived the study, designed the experiments, and wrote the manuscript. All the authors discussed the results and commented on the manuscript.

DECLARATION OF INTERESTS

The authors declare no competing interests.

Received: June 27, 2019
Revised: September 4, 2020
Accepted: October 7, 2020
Published: November 5, 2020

REFERENCES

1. Su, S.H., Gibbs, N.M., Jancewicz, A.L., and Masson, P.H. (2017). Molecular mechanisms of root gravitropism. Curr. Biol. *27*, R964–R972.
2. Armengot, L., Marquès-Bueno, M.M., and Jaillais, Y. (2016). Regulation of polar auxin transport by protein and lipid kinases. J. Exp. Bot. *67*, 4015–4037.
3. Rakusová, H., Fendrych, M., and Friml, J. (2015). Intracellular trafficking and PIN-mediated cell polarity during tropic responses in plants. Curr. Opin. Plant Biol. *23*, 116–123.
4. Adamowski, M., and Friml, J. (2015). PIN-dependent auxin transport: action, regulation, and evolution. Plant Cell *27*, 20–32.
5. Zhang, Y., Xiao, G., Wang, X., Zhang, X., and Friml, J. (2019). Evolution of fast root gravitropism in seed plants. Nat. Commun. *10*, 3480.
6. Retzer, K., Akhmanova, M., Konstantinova, N., Malínská, K., Leitner, J., Petrášek, J., and Luschnig, C. (2019). Brassinosteroid signaling delimits root gravitropism via sorting of the *Arabidopsis* PIN2 auxin transporter. Nat. Commun. *10*, 5516.
7. Xu, T., Dai, N., Chen, J., Nagawa, S., Cao, M., Li, H., Zhou, Z., Chen, X., De Rycke, R., Rakusová, H., et al. (2014). Cell surface ABP1-TMK auxin-sensing complex activates ROP GTPase signaling. Science *343*, 1025–1028.
8. Lin, D., Nagawa, S., Chen, J., Cao, L., Chen, X., Xu, T., Li, H., Dhonukshe, P., Yamamuro, C., Friml, J., et al. (2012). A ROP GTPase-dependent auxin signaling pathway regulates the subcellular distribution of PIN2 in *Arabidopsis* roots. Curr. Biol. *22*, 1319–1325.
9. Chen, X., Naramoto, S., Robert, S., Tejos, R., Löfke, C., Lin, D., Yang, Z., and Friml, J. (2012). ABP1 and ROP6 GTPase signaling regulate clathrin-mediated endocytosis in *Arabidopsis* roots. Curr. Biol. *22*, 1326–1332.
10. Xu, T., Wen, M., Nagawa, S., Fu, Y., Chen, J.G., Wu, M.J., Perrot-Rechenmann, C., Friml, J., Jones, A.M., and Yang, Z. (2010). Cell surface- and Rho GTPase-based auxin signaling controls cellular interdigitation in *Arabidopsis*. Cell *143*, 99–110.
11. De Smet, I., Voss, U., Jürgens, G., and Beeckman, T. (2009). Receptor-like kinases shape the plant. Nat. Cell Biol. *11*, 1166–1173.
12. Neben, C.L., Lo, M., Jura, N., and Klein, O.D. (2019). Feedback regulation of RTK signaling in development. Dev. Biol. *447*, 71–89.
13. Belkhadir, Y., and Jaillais, Y. (2015). The molecular circuitry of brassinosteroid signaling. New Phytol. *206*, 522–540.
14. Jaillais, Y., Hothorn, M., Belkhadir, Y., Dabi, T., Nimchuk, Z.L., Meyerowitz, E.M., and Chory, J. (2011). Tyrosine phosphorylation controls brassinosteroid receptor activation by triggering membrane release of its kinase inhibitor. Genes Dev. *25*, 232–237.
15. Wang, X., and Chory, J. (2006). Brassinosteroids regulate dissociation of BKI1, a negative regulator of BRI1 signaling, from the plasma membrane. Science *313*, 1118–1122.
16. Wang, J., Jiang, J., Wang, J., Chen, L., Fan, S.L., Wu, J.W., Wang, X., and Wang, Z.X. (2014). Structural insights into the negative regulation of BRI1 signaling by BRI1-interacting protein BKI1. Cell Res. *24*, 1328–1341.
17. Simon, M.L., Platre, M.P., Marquès-Bueno, M.M., Armengot, L., Stanislas, T., Bayle, V., Caillaud, M.C., and Jaillais, Y. (2016). A PtdIns(4)P-driven

electrostatic field controls cell membrane identity and signalling in plants. Nat. Plants *2*, 16089.

18. Boutté, Y., and Jaillais, Y. (2020). Metabolic cellular communications: feedback mechanisms between membrane lipid homeostasis and plant development. Dev. Cell *54*, 171–182.
19. Jaillais, Y., and Vert, G. (2016). Brassinosteroid signaling and BRI1 dynamics went underground. Curr. Opin. Plant Biol. *33*, 92–100.
20. Kang, Y.H., and Hardtke, C.S. (2016). *Arabidopsis* MAKR5 is a positive effector of BAM3-dependent CLE45 signaling. EMBO Rep. *17*, 1145–1154.
21. Xuan, W., Audenaert, D., Parizot, B., Möller, B.K., Njo, M.F., De Rybel, B., De Rop, G., Van Isterdael, G., Mähönen, A.P., Vanneste, S., and Beeckman, T. (2015). Root cap-derived auxin pre-patterns the longitudinal axis of the *Arabidopsis* root. Curr. Biol. *25*, 1381–1388.
22. Hazak, O., Brandt, B., Cattaneo, P., Santiago, J., Rodriguez-Villalon, A., Hothorn, M., and Hardtke, C.S. (2017). Perception of root-active CLE peptides requires CORYNE function in the phloem vasculature. EMBO Rep. *18*, 1367–1381.
23. Abas, L., Benjamins, R., Malenica, N., Paciorek, T., Wiśniewska, J., Moulinier-Anzola, J.C., Sieberer, T., Friml, J., and Luschnig, C. (2006). Intracellular trafficking and proteolysis of the *Arabidopsis* auxin-efflux facilitator PIN2 are involved in root gravitropism. Nat. Cell Biol. *8*, 249–256.
24. Zhang, X., Adamowski, M., Marhava, P., Tan, S., Zhang, Y., Rodriguez, L., Zwiewka, M., Pukyšová, V., Sánchez, A.S., Raxwal, V.K., et al. (2020). *Arabidopsis* flippases cooperate with ARF GTPase exchange factors to regulate the trafficking and polarity of PIN auxin transporters. Plant Cell *32*, 1644–1664.
25. Platre, M.P., Bayle, V., Armengot, L., Bareille, J., Marquès-Bueno, M.D.M., Creff, A., Maneta-Peyret, L., Fiche, J.B., Nollmann, M., Miège, C., et al. (2019). Developmental control of plant Rho GTPase nano-organization by the lipid phosphatidylserine. Science *364*, 57–62.
26. Poraty-Gavra, L., Zimmermann, P., Haigis, S., Bednarek, P., Hazak, O., Stelmakh, O.R., Sadot, E., Schulze-Lefert, P., Gruissem, W., and Yalovsky, S. (2013). The *Arabidopsis* Rho of plants GTPase AtROP6 functions in developmental and pathogen response pathways. Plant Physiol. *161*, 1172–1188.
27. Sorek, N., Segev, O., Gutman, O., Bar, E., Richter, S., Poraty, L., Hirsch, J.A., Henis, Y.I., Lewinsohn, E., Jürgens, G., and Yalovsky, S. (2010). An *S*-acylation switch of conserved G domain cysteines is required for polarity signaling by ROP GTPases. Curr. Biol. *20*, 914–920.
28. Xuan, W., Band, L.R., Kumpf, R.P., Van Damme, D., Parizot, B., De Rop, G., Opdenacker, D., Möller, B.K., Skorzinski, N., Njo, M.F., et al. (2016). Cyclic programmed cell death stimulates hormone signaling and root development in *Arabidopsis*. Science *351*, 384–387.
29. Baster, P., Robert, S., Kleine-Vehn, J., Vanneste, S., Kania, U., Grunewald, W., De Rybel, B., Beeckman, T., and Friml, J. (2013). SCF(TIR1/AFB)-auxin signalling regulates PIN vacuolar trafficking and auxin fluxes during root gravitropism. EMBO J. *32*, 260–274.
30. Paciorek, T., Zazímalová, E., Ruthardt, N., Petrásek, J., Stierhof, Y.D., Kleine-Vehn, J., Morris, D.A., Emans, N., Jürgens, G., Geldner, N., and Friml, J. (2005). Auxin inhibits endocytosis and promotes its own efflux from cells. Nature *435*, 1251–1256.
31. Pan, X., Fang, L., Liu, J., Senay-Aras, B., Lin, W., Zheng, S., Zhang, T., Guo, J., Manor, U., Van Norman, J., et al. (2020). Auxin-induced signaling protein nanoclustering contributes to cell polarity formation. Nat. Commun. *11*, 3914.
32. Cao, M., Chen, R., Li, P., Yu, Y., Zheng, R., Ge, D., Zheng, W., Wang, X., Gu, Y., Gelová, Z., et al. (2019). TMK1-mediated auxin signalling regulates differential growth of the apical hook. Nature *568*, 240–243.
33. Dai, N., Wang, W., Patterson, S.E., and Bleecker, A.B. (2013). The TMK subfamily of receptor-like kinases in *Arabidopsis* display an essential role in growth and a reduced sensitivity to auxin. PLoS ONE *8*, e60990.
34. Yazaki, J., Galli, M., Kim, A.Y., and Ecker, J.R. (2018). Profiling interactome networks with the HaloTag-NAPPA *in situ* protein array. Curr. Protoc. Plant Biol. *3*, e20071.
35. Fendrych, M., Akhmanova, M., Merrin, J., Glanc, M., Hagihara, S., Takahashi, K., Uchida, N., Torii, K.U., and Friml, J. (2018). Rapid and reversible root growth inhibition by TIR1 auxin signalling. Nat. Plants *4*, 453–459.
36. Finet, C., and Jaillais, Y. (2012). Auxology: when auxin meets plant evo-devo. Dev. Biol. *369*, 19–31.
37. Robert, S., Kleine-Vehn, J., Barbez, E., Sauer, M., Paciorek, T., Baster, P., Vanneste, S., Zhang, J., Simon, S., Čovanová, M., et al. (2010). ABP1 mediates auxin inhibition of clathrin-dependent endocytosis in *Arabidopsis*. Cell *143*, 111–121.
38. Jaillais, Y., Belkhadir, Y., Balsemão-Pires, E., Dangl, J.L., and Chory, J. (2011). Extracellular leucine-rich repeats as a platform for receptor/coreceptor complex formation. Proc. Natl. Acad. Sci. USA *108*, 8503–8507.
39. Ulmasov, T., Murfett, J., Hagen, G., and Guilfoyle, T.J. (1997). Aux/IAA proteins repress expression of reporter genes containing natural and highly active synthetic auxin response elements. Plant Cell *9*, 1963–1971.
40. Benková, E., Michniewicz, M., Sauer, M., Teichmann, T., Seifertová, D., Jürgens, G., and Friml, J. (2003). Local, efflux-dependent auxin gradients as a common module for plant organ formation. Cell *115*, 591–602.
41. Xu, J., and Scheres, B. (2005). Dissection of *Arabidopsis* ADP-RIBOSYLATION FACTOR 1 function in epidermal cell polarity. Plant Cell *17*, 525–536.
42. Karimi, M., Bleys, A., Vanderhaeghen, R., and Hilson, P. (2007). Building blocks for plant gene assembly. Plant Physiol. *145*, 1183–1191.
43. Curtis, M.D., and Grossniklaus, U. (2003). A Gateway cloning vector set for high-throughput functional analysis of genes *in planta*. Plant Physiol. *133*, 462–469.
44. Nito, K., Wong, C.C., Yates, J.R., III, and Chory, J. (2013). Tyrosine phosphorylation regulates the activity of phytochrome photoreceptors. Cell Rep. *3*, 1970–1979.
45. Yazaki, J., Galli, M., Kim, A.Y., Nito, K., Aleman, F., Chang, K.N., Carvunis, A.R., Quan, R., Nguyen, H., Song, L., et al. (2016). Mapping transcription factor interactome networks using HaloTag protein arrays. Proc. Natl. Acad. Sci. USA *113*, E4238–E4247.
46. Marquès-Bueno, M.M., Morao, A.K., Cayrel, A., Platre, M.P., Barberon, M., Caillieux, E., Colot, V., Jaillais, Y., Roudier, F., and Vert, G. (2016). A versatile multisite gateway-compatible promoter and transgenic line collection for cell type-specific functional genomics in *Arabidopsis*. Plant J. *85*, 320–333.
47. Mitra, S.K., Chen, R., Dhandaydham, M., Wang, X., Blackburn, R.K., Kota, U., Goshe, M.B., Schwartz, D., Huber, S.C., and Clouse, S.D. (2015). An autophosphorylation site database for leucine-rich repeat receptor-like kinases in *Arabidopsis thaliana*. Plant J. *82*, 1042–1060.
48. Schneider, C.A., Rasband, W.S., and Eliceiri, K.W. (2012). NIH Image to ImageJ: 25 years of image analysis. Nat. Methods *9*, 671–675.
49. Shaner, N.C., Lambert, G.G., Chammas, A., Ni, Y., Cranfill, P.J., Baird, M.A., Sell, B.R., Allen, J.R., Day, R.N., Israelsson, M., et al. (2013). A bright monomeric green fluorescent protein derived from *Branchiostoma lanceolatum*. Nat. Methods *10*, 407–409.
50. Brunoud, G., Wells, D.M., Oliva, M., Larrieu, A., Mirabet, V., Burrow, A.H., Beeckman, T., Kepinski, S., Traas, J., Bennett, M.J., and Vernoux, T. (2012). A novel sensor to map auxin response and distribution at high spatio-temporal resolution. Nature *482*, 103–106.
51. Simon, M.L., Platre, M.P., Assil, S., van Wijk, R., Chen, W.Y., Chory, J., Dreux, M., Munnik, T., and Jaillais, Y. (2014). A multi-colour/multi-affinity

CellPress
OPEN ACCESS

marker set to visualize phosphoinositide dynamics in *Arabidopsis*. Plant J. *77*, 322–337.

52. Wang, Z.P., Xing, H.L., Dong, L., Zhang, H.Y., Han, C.Y., Wang, X.C., and Chen, Q.J. (2015). Egg cell-specific promoter-controlled CRISPR/Cas9 efficiently generates homozygous mutants for multiple target genes in *Arabidopsis* in a single generation. Genome Biol. *16*, 144.

53. Grabov, A., Ashley, M.K., Rigas, S., Hatzopoulos, P., Dolan, L., and Vicente-Agullo, F. (2005). Morphometric analysis of root shape. New Phytol. *165*, 641–651.

54. Livak, K.J., and Schmittgen, T.D. (2001). Analysis of relative gene expression data using real-time quantitative PCR and the 2(−Delta Delta C(T)) method. Methods *25*, 402–408.

55. von Wangenheim, D., Hauschild, R., Fendrych, M., Barone, V., Benková, E., and Friml, J. (2017). Live tracking of moving samples in confocal microscopy for vertically grown roots. eLife *6*, e26792.

56. Grones, P., Chen, X., Simon, S., Kaufmann, W.A., De Rycke, R., Nodzyński, T., Zažímalová, E., and Friml, J. (2015). Auxin-binding pocket of ABP1 is crucial for its gain-of-function cellular and developmental roles. J. Exp. Bot. *66*, 5055–5065.

STAR★METHODS

KEY RESOURCES TABLE

REAGENT or RESOURCE	SOURCE	IDENTIFIER
**Antibodies**		
Mouse monoclonal anti-Flag clone M2	Sigma-Aldrich	Cat#1804; RRID: AB_262044
Mouse monoclonal anti-HA clone 12CA5	Sigma-Aldrich	Cat#11583816001; RRID: AB_514505
Mouse monoclonal anti-GFP HRP clone GG4-2C2.12.10	Miltenyi Biotec	Cat#130-091-833; RRID: AB_247003
Rat monoclonal anti-HA High Affinity clone 3F10	Sigma-Aldrich	Cat#11867423001; RRID: AB_390918
**Bacterial and Virus Strains**		
Agrobacteirum tumefaciens C58 GV3101	N/A	N/A
Escherichia coli DH5alpa	N/A	N/A
Escherichia coli BL21 (DE3) Rosetta 2 Novagen	Sigma-Aldrich	Cat#69450
**Chemicals, Peptides, and Recombinant Proteins**		
Cycloheximide (CHX)	Sigma-Aldrich	Cat#C1988
MG-132	VWR	Cat#474790-5
NAA	Sigma-Aldrich	Cat#N0640
IAA	Sigma-Aldrich	Cat#I2886
5-F-IAA	Goldbiotech	Cat#F-160-5
PEO-IAA	Sigma-Aldrich	Cat#CDS013406
Brassinolide	Sigma-Aldrich	Cat#B1439
Benzoid Acid (BA)	Sigma-Aldrich	Cat#242381
**Experimental Models: Cell Lines**		
Arabidopsis suspension culture (PSB-L)	N/A	N/A
**Experimental Models: Organisms/Strains**		
Arabidopsis: MAKR2-Ox1 (2x35Sprom::MAKR2-2xmCHERRY)	This manuscript	NASC #N2110134
Arabidopsis: MAKR2-Ox2 (2x35Sprom::MAKR2-mCITRINE)	[17]	NASC #N2110135
Arabidopsis: amiMAKR2	This manuscript	NASC #N2110142 and #N2110143
Arabidopsis: DR5::GUS	[39]	N/A
Arabidopsis: DR5rev::GFP	[40]	NASC #N9361
Arabidopsis: DR5::GUS;MAKR2-Ox1	This manuscript	N/A
Arabidopsis: DR5rev::GFP;MAKR2-Ox1	This manuscript	N/A
Arabidopsis: makr2-1 (crispr)	This manuscript	NASC #N2110144
Arabidopsis: MAKR2prom::MAKR2-tdYFP	This manuscript	NASC #N2110140
Arabidopsis: MAKR2prom::MAKR2-GUS	This manuscript	N/A
Arabidopsis: MAKR2prom::VENUS^{NLS}	This manuscript	NASC #N2110141
Arabidopsis: MAKR2prom::MAKR2-2xmCHERRY	This manuscript	N/A
Arabidopsis: PIN2prom::PIN2-GFP	[41]	N/A
Arabidopsis: PIN2prom::PIN2-GFP;MAKR2-Ox1	This manuscript	N/A
Arabidopsis: PIN2prom::PIN2-GFP;amiMAKR2	This manuscript	N/A
Arabidopsis: ROP6prom::mCITRINE-ROP6 in *rop6-2*	[25]	NASC #N2109740

(*Continued on next page*)

Continued

REAGENT or RESOURCE	SOURCE	IDENTIFIER
Arabidopsis: ROP6-Ox	[27]	N/A
Arabidopsis: MAKR2-Ox1;ROP6-Ox	This manuscript	N/A
Arabidopsis: TMK1-Ox (UBQ10prom::TMK1-2xmCHERRY)	This manuscript	NASC #N2110145
Arabidopsis: MAKR2-Ox2;TMK1-Ox	This manuscript	N/A
Arabidopsis: tmk1-1;tmk4	[33]	SALK_01360 Wiscseq_DsLox377-380D21.1
Arabidopsis: TMK1prom::2xmCHERRY^{NLS}	This manuscript	N/A
Arabidopsis: MAKR2-Ox2;TMK1^{K616R}-Ox	This manuscript	N/A
Arabidopsis: MAKR2-mCit^{GEGE}	This manuscript	NASC #N2110136 and #N2110137
Arabidopsis: MAKR2^{11Q}-mCit	This manuscript	NASC #N2110138 and #N2110139
Oligonucleotides		
Primer: MAKR2prom-topo F GTCTCTTTCAGTCATACCTCTCTCTAA	This manuscript	N/A
Primer: MAKR2prom-topo R TGTTGGGAAAGCCAGAATCA	This manuscript	N/A
Primer: MAKR2-B1 GGGGACAAGTTTGTACAAAA AAGCAGGCTTAACCATGGAAGCTT TCAGTCTCCTTAAC	This manuscript	N/A
Primer: MAKR-B2 GGGGACCACTTTGT ACAAGAAAGCTGGGTATTTCTCGTAA GAGGAAGGTTCACT	This manuscript	N/A
Primer: tdYFP-B2R GGGGACAGCTTTCTTG TACAAAGTGGCTATGGTATCCAAAGGT GAAGAAGACA	This manuscript	N/A
Primer: tdYFP-B3 GGGGACAACTTTGTATAAT AAAGTTGCTCACTTATACAGTTCGT CCATCCCC	This manuscript	N/A
Primer: TMK1-B1 F GGGGACAAGTTT GTACAAAAAAGCAGGCTTAA CCATGAAGAAAAGAA GAACCTTTCTTCT	This manuscript	N/A
Primer: TMK1-B2 R GGGGACCACTTTG TACAAGAAAGCTGGGT ATCGTCCATCTACT GAAGTGAATGACT	This manuscript	N/A
Primer: tdYFP-B2R GGGGACAGCTTTCTTGTA CAAAGTGGCTATGGTAT CCAAAGGTGAAGAAGACA	This manuscript	N/A
Primer: tdYFP-B3 GGGGACAACTTTGTA TAATAAAGTTGCTCACTTATACAGTTCG TCCATCCCC	This manuscript	N/A
Primer: TMK1prom-Fw GTATAGAAAAG TTGCTGTGGAATTTTA ATCTTAATTAAGGGAAGC	This manuscript	N/A
Primer: TMK1prom-Rev TTTTTTGT ACAAACTTGCAGCTTGA AGAAACAGAGGATTGAAGAAGAAACAG	This manuscript	N/A
Primer: p5′-open-Fw CCTCTGTTTCTTCAAGCTGCAAGTTT GTACAAAAAAGTTGAACG	This manuscript	N/A

(*Continued on next page*)

Continued

REAGENT or RESOURCE	SOURCE	IDENTIFIER
Primer: p5′-open-Rev TTAAGATTAAAATTCCACAGCAACTTTT CTATACAAAGTTGG	This manuscript	N/A
Primer: TMK1^{KD}(K616R)-F ATTGCGGTTAGGAGAATGGAGAATGGA GTTATTGCTGG	This manuscript	N/A
Primer: TMK1^{KD}(K616R)-R CCATTCTCCATTCTCCTAACCGCAAT CTTCGTTCCATC	This manuscript	N/A
Primer: TMK1-FL-B1 GGGGACAAGTTTG TACAAAAAAGCAGGCTCAAT GAAGAAAAGAAGAACCTTTC	This manuscript	N/A
Primer: TMK1-FL-B2-R GGGGACCAC TTTGTACAAGAAAGCTG GGTCTCGTCCATCTACTGAAGTGAAT	This manuscript	N/A
Primer: TMK1-ΔKD-B2-R GGGGACCAC TTTGTACAAGAAAGCTGGGTCGTTGTT AGTCACAGAACGAAG	This manuscript	N/A
Primer: F1 gRNA3 ATATATGGTCTCGATTGCTTTCAGTCTC CTTAACTACGTT	This manuscript	N/A
Primer: F0 gRNA3 TGCTTTCAGTCTCCTTAACTACGTTTTA GAGCTAGAAATAGC	This manuscript	N/A
Primer: R0 gRNA4 AACGCGGAGTAACGGCAAAGAGACA ATCTCTTAGTCGACTCTAC	This manuscript	N/A
Primer: R2 gRNA4 ATTATTGGTCTCGAAACGCGGAGTAAC GGCAAAGAGACAA	This manuscript	N/A
Primer: MAKR2 qPCR F CAACAAGATAGTATTCAAAGTGCCA	This manuscript	N/A
Primer: MAKR2 qPCR R GAGGAAGGTTCACTCACCGA	This manuscript	N/A
Primer: EF1 alpha qPCR F TGAGCACGCTCTTCTTGCTTTCA	This manuscript	N/A
Primer: EF1 alpha qPCR R GGTGGTGGCATCCATCTTGTTACA	This manuscript	N/A
Primer: GAPDH F GAATCCGAAGGCAAACTCAA	This manuscript	N/A
Primer: GAPDH R AAACTTGTCGCTCAATGCAA	This manuscript	N/A
Recombinant DNA		
Empty gateway destination vector: *pB7m34GW*	[42]	N/A
Empty gateway destination vector: *pH7m34GW*	[42]	N/A
Empty gateway destination vector: *pLOK180_FR7m34GW*	Gift from Lothar Kalmbach, Cambridge	N/A
Empty gateway destination vector: *pMDC32*	[43]	N/A
Empty gateway destination vector: *pTNT-HA-GW*	[44]	N/A

(*Continued on next page*)

CellPress
OPEN ACCESS

Continued

REAGENT or RESOURCE	SOURCE	IDENTIFIER
Empty gateway destination vector: pIX:HALO:ccdB-CmR	[45]	ABRC #CD3-1742
Expression plasmid: pET28-duet	This manuscript	N/A
Entry gateway vector: *2x35prom/pDONR4-P1R*	[46]	NASC #N2106316
Entry gateway vector: *UBQ10prom/pDONR4-P1R*	[46]	NASC #N2106315
Entry gateway vector: *MAKR2prom/pENTR5′*	This manuscript	N/A
Entry gateway vector: *TMK1prom/pDONRP4-P1R*	This manuscript	N/A
Entry gateway vector: MAKR2gnoSTOP/pDONR221	[17]	N/A
Entry gateway vector: MAKR2wSTOP/pDONR221	This manuscript	N/A
Entry gateway vector: TMK1noSTOP/pDONR221	This manuscript	N/A
Entry gateway vector: TMK1^{K616R}noSTOP/pDONR221	This manuscript	N/A
Entry gateway vector: MAKR2(CDS)noSTOP/pDONR221	This manuscript	N/A
Entry gateway vector: MAKR2^{11Q}noSTOP/pDONR221	This manuscript	N/A
Entry gateway vector: 2xmCHERRY-4xMyc/pDONRP2R-P3	[14]	NASC #N2106292
Entry gateway vector: mCITRINE/pDONRP2R-P3	[14]	NASC #N2106288
Entry gateway vector: tdYFP/pDONRP2RP3	This manuscript	N/A
Entry gateway vector: mCITRINE^{GEGE}/pDONRP2R-P3	[17]	N/A
Entry gateway vector: TMK1^{cyt}/pDONR221	This manuscript	N/A
Entry gateway vector: HSL1^{cyt}/pDONR221	[47]	ABRC #N1g28440Ze_K
Entry gateway vector: mCITRINEwSTOP/pDONR221	[46]	N/A
Entry gateway vector: TMK1$^{\Delta kinase}$/pDONR221	This manuscript	N/A
Destination expression vector: TMK1^{cyt}/pTNT-HA-GW	This manuscript	N/A
Destination expression vector: HSL1^{cyt}/pTNT-HA-GW	This manuscript	N/A
Destination expression vector: mCITRINE/pIX-HA-GW	This manuscript	N/A
Destination expression vector: MAKR2/pIX-HA-GW	This manuscript	N/A
Expression vector: MAKR2-TMK1^{cyt}/pET28-duet	This manuscript	N/A
Expression vector: MAKR2-TMK1^{cytK616R}/pET28-duet	This manuscript	N/A
Destination vector: *UBQ10prom::TMK1-3HA/pB7m34GW*	This manuscript	N/A
Destination vector: *UBQ10prom::TMK1$^{\Delta kinase}$-3HA/pB7m34GW*	This manuscript	N/A

(*Continued on next page*)

Continued

REAGENT or RESOURCE	SOURCE	IDENTIFIER
Destination vector: *2x35Sprom::MAKR2-2xmCHERRY/pH7m34GW*	This manuscript	N/A
Destination vector: *2x35Sprom::MAKR2-mCitrine/pB7m34GW*	This manuscript	N/A
Destination vector: *amiMAKR2/pMDC32*	This manuscript	N/A
Destination vector: *amiMAKR2/pB7WG2*	This manuscript	N/A
Destination vector: *MAKR2prom::MAKR2-2xmCHERRY/pH7m34GW*	This manuscript	N/A
Destination vector: *MAKR2prom::MAKR2-tdYFP/pB7m34GW*	This manuscript	N/A
Destination vector: *UBQ10prom::TMK1-2xmCHERRY-/pH7m34GW*	This manuscript	N/A
Destination vector: *UBQ10prom::TMK1^{K616R}-2xmCHERRY-/pH7m34GW*	This manuscript	N/A
Destination vector: *2x35S::MAKR2-mCit^{GEGE}/pB7m34GW*	This manuscript	N/A
Destination plasmid: *2x35S::MAKR2^{11Q}-mCit/pLOK180_pFR7m34GW*	This manuscript	N/A
Destination vector: *MAKR2prom::MAKR2-GUS*	This manuscript	N/A
Destination vector: MAKR2prom::VENUS^{NLS}	This manuscript	N/A
Destination vector: TMK1prom::2xmCHERRY^{NLS}	This manuscript	N/A
Software and Algorithms		
ImageJ	NIH[48]	https://imagej.nih.gov/ij/
R	R project	https://www.r-project.org/
Excel	Microsoft	https://www.microsoft.com/
Other		
Plant/Fungi RNA Purification Kit	Sigma-Aldrich	Cat#E4813
Reverse transcriptase SuperScript IV VILO Master Mix	Thermofisher	Cat#11756050
Gateway BP clonase	Thermofisher	Cat#11789100
Gateway LRII plus clonase	Thermofisher	Cat#12538120
TnT Coupled Wheat Germ Extract System	Promega / Thermofisher	Cat#L5030
Halo-tag magnetic beads	Promega / Thermofisher	Cat#7281

RESOURCE AVAILABILITY

Lead Contact

Further information and requests for resources and reagents should be directed to and will be fulfilled by the Lead Contact, Yvon Jaillais (yvon.jaillais@ens-lyon.fr).

Materials Availability

There is no restriction of the material generated in this study (plasmids and *Arabidopsis* transgenic seeds).

Data and Code Availability

This study did not generate any unique datasets or code.

EXPERIMENTAL MODEL AND SUBJECT DETAILS

Plant material and Growth conditions

In all the experiments, wild-type Col-0 ecotype of *Arabidopsis thaliana* was used as a control and all transgenic lines were produced in Col-0 background. Plants were grown in continuous light on MS supplemented with vitamins (Duchefa) and 1% sucrose. The following transgenic lines have been described before: *35S::GFP-ROP6 (ROP6-Ox);*[27] *DR5prom::GUS;*[39] *DR5rev-prom::GFP;*[40] *PIN2::PIN2-GFP;*[41] *ROP6prom::mCitrine-ROP6g/rop6-2,*[25] *tmk1-1* (SALK_016360) and *tmk1tmk4* mutants (*tmk4*: Wiscseq_DsLox377-380D21.1).[33]

Plant Transformation and Selection

Each construct was transformed into C58 GV3101 *Agrobacterium tumefaciens* strain and selected on YEB media (5g/L beef extract; 1g/L yeast extract; 5g/L peptone; 5g/L sucrose; 15 g/L bactoagar; pH 7.2) supplemented with antibiotics (Spectinomycin, Gentamycin). After two days of growth at 28°C, bacteria were collected using a single-use cell scraper, re-suspended in about 200 mL of transformation buffer (10mM MgCl2; 5% sucrose; 0.25% silweet) and plants were transformed by dipping. Plants from the Columbia–0 (Col0) accession were used for transformation.

Primary transformants (T1) were selected *in vitro* on the appropriate antibiotic/herbicide (glufosinate for mCITRINE, hygromycin for mCHERRY-tagged proteins or using the FastRed screening method). Approximately 20 independent T1s were selected for each line. In the T2 generation at least 3 independent transgenic lines were selected using the following criteria when possible: i) good expression level in the root for detection by confocal microscopy, ii) uniform expression pattern, iii) single insertion line (1 sensitive to 3 resistant segregation ratio). Lines were rescreened in T3 using similar criteria as in T2 with the exception that we selected homozygous lines (100% resistant/fluorescent). At this step, we selected one to three transgenic line(s) that was(were) used for further analyses and crosses.

METHOD DETAILS

Cloning and characterization of transgenic lines

The cloning for the transgenic lines production was performed using the multi-site gateway system (thermofisher).

Cloning of promoters into gateway entry vectors

The *MAKR2* promoter was amplified from Col-0 genomic DNA using *MAKR2prom-topoF/MAKR2prom-topoF* (Key Resources Table) and cloned into the *pENTR 5′-TOPO TA* vector by TOPO TA Cloning (thermofisher) to give *MAKR2prom/pENTR5′*. The *TMK1* promoter was amplified from Col-0 genomic DNA using TMK1prom-Fw and TMK1prom-Rev and cloned into pDONRP4-P1R using Gibson cloning, with pDONRP4-P1R having been amplified using the p5′-open-Fw and p5′-open-rev primers, to give TMK1prom/pDONRP4-P1R. *2x35Sprom/pDONRP4P1R* and *UBQ10prom/pDONRP4P1R* were described previously.[14,46]

Cloning of genes into gateway entry vectors

The *MAKR2* genomic fragment was amplified from Col-0 genomic DNA using *MAKR2-B1/MAKR2-B2* primers and introduced into the *pDONR221* by BP recombination to give *MAKR2gnoSTOP/pDONR221*. *TMK1* was amplified from Col-0 cDNA using *TMK1-B1/TMK1-B2* primers and recombined into *pDONR221* by BP cloning to give *TMK1noSTOP/pDONR221*. *MAKR2(CDS)noSTOP/pDONR221* was previously described.[17] *TMK1$^{KD(K616R)}$noSTOP/pDONR221* was obtained by site directed mutagenesis by amplifying *TMK1noSTOP/pDONR221* with the TMK1^{KD}-K616F/TMK1^{KD}-K616R primer pair.

The MAKR2^{11Q} (CDS) no STOP sequence was synthesized by IDT technologies in the pUCIDT-AMP vector and subsequently recombined into pDONR221 by BP reaction to obtain *MAKR2^{11Q} (CDS) no STOP/pDONR221*. In the MAKR2^{11Q} sequence, the 11 positively charged residues (lysine -K- or arginine -R-) of the cationic region were mutated to the neutral amino acid glutamine (Q) as follows:

```
> MAKR2
780 gag aaa cga ttc gtg atg atg caa aag tac tta aag aag gta aaa cca ctt tac atc aga
     E   K   R   F   V   M   M   Q   K   Y   L   K   K   V   K   P   L   Y   I   R
gtt tca cgt cgt tac ggc gag aaa tta cga cac 870
 V   S   R   R   Y   G   E   K   L   R   H
> MAKR2^11Q
780 gag caa caa ttc gtg atg atg caa cag tac tta cag cag gta caa cca ctt tac atc caa
     E   Q   Q   F   V   M   M   Q   Q   Y   L   Q   Q   V   Q   P   L   Y   I   Q
gtt tca caa cag tac ggc gag caa tta caa cac 870
 V   S   Q   Q   Y   G   E   Q   L   Q   H
```

Cloning of artificial microRNAs into gateway entry vectors

Artificial microRNAs were designed using web microRNA designer (http://wmd3.weigelworld.org/cgi-bin/webapp.cgi). The following sequence was synthesized by IDT technologies and subsequently recombined into pDONR/Zeo by BP recombination to give *ami-MAKR2/* pDONR/Zeo:

> amiMAKR2

acaagtttgtacaaaaaagcaggctcaaacacacgctcggacgcatattacacatgttcatacacttaatactcgctgttttgaattgatgttttaggaatatatatgt**aga**tatgtaaa
gtggttttaccta**tcacaggtcgtgatatgattcaattagcttccgactcattcatccaaataccgagtcgccaaaattcaaactagactcgttaaatgaatgaatgatgcg**
gtagacaaattggatcattgattctctttgataggtaaaaccactttacata**tct**ctcttttgtattccaattttcttgattaatctttcctgcacaaaaacatgcttgatccactaagtga
catatatgctgccttcgtatatatagttctggtaaaattaacattttgggtttatctttatttaaggcatcgccatgacccagctttcttgtacaaagtggt

Attb1 and Attb2 for gateway cloning
amiMAKR2/ pDONR/Zeo was transferred by LR recombination into pMDC32[43] or pB7WG2.[42]

Cloning of reporters into gateway entry vectors:

tdYFP is a tandem dimer of dlanYFP (from *Branchiostoma lanceolatum*).[49] The following sequence was codon optimized for *Arabidopsis* and synthetized by IDT (https://eu.idtdna.com/site):

> tdYFP:

atggtatccaaaggtgaagaagacaatatggcatcattacctgcaacccatgagcttcacatctttggtt
cgtttaatggagtagattttgatatggttggaagagggacaggtaaccctaacgacggttatgaagaact
taaccttaaatctaccaagggagatctccaattctctccttggattttggtcccacagattggttatggt
tttcatcagtatttgccatttcccgacggaatgtctccattccaagcagccatgaaagacggctcgggct
accaggttcacagaacaatgcaatttgaagatggtgcaagtctcacttctaactatcgttatacgtatga
aggaagtcatattaaaggcgaatttcaggtaaagggaactggatttcctgccgacggaccagttatgaca
aattcattgactgcagcagattggtgtgtgacaaagatgttatatccaaacgacaagactatcatctcaa
cttttgattggacttacactacgggtaatggaaaaaggtatcaaagcacggcaagaaccacttacacatt
cgccaagccaatggcagcaaacatactaaagaaccagccaatgttcgtttttagaaagacagagctcaag
cattcaaagactgaacttaacttcaaagaatggcaaaaagcattcaccgatgtgatgggtcatggaactg
gatctacgggatctggctcaagtggaacagcaagttctgaagataatatggcatcgctgcctgcaac
ccatgaacttcatatatttgggtctttcaatggagtcgattttgacatggtaggacgaggtacaggtaat
cctaacgatgggtacgaggagttgaacctaaagagtactaagggagacctccagttcagtccctggattt
tagtaccgcaaatcggttatggattccaccagtatttaccatttccggacggaatgtcgccctttcaggc
ggcgatgaaagacggctctggatatcaggttcatagaaccatgcaatttgaggacggagcatctctgacg
tccaactatagatatacttatgaaggctcgcacattaaaggagagtttcaggtgaagggaactggattcc
ctgctgacggccctgtcatgacaaatagccttactgctgcggattggtgtgttaccaaaatgctctaccc
taatgacaagactatcatcagtacttttgactggacttatactactggaaacgggaagcgatatcagtcc
acggcaagaactacatacacctttgccaaacctatggcagccaacatcttgaaaaaccaaccaatgtttg
tgttcaggaagactgaacttaaacactcaaaaaccgaactgaatttcaaagagtggcagaaagctttcac
agatgttatggggatggacgaactgtataagtga

The synthetic tdYFP gene was subsequently amplified using the tdYFP-B2R/tdYFP-B3 primer pair and cloned into pDONRP2R-P3 using BP recombination. mCITRINE/pDONRP2RP3, mCITRINEnoSTOP/pDONR221, mCITRINE^{GEGE}/pDONRP2RP3, 2xmCHERRY-4xMyc/pDONRP2RP3, GUS/pDONRP2R-P3, VENUS^{NLS}/pDONR221 and mock/pDONRP2RP3 were described previously.[14,17,46,50]

All primers used for cloning are indicated in the Key Resources Table.

Destination vectors and plant transformation

Arabidopsis stable transformation and selection were performed as described.[51] Final destination vectors for plant transformation were obtained using the LR recombination system (http://www.thermofisher.com/) using the *pB7m34GW*[42] (basta resistant), *pH7m34GW*[42] (hygromycin resistant), *pB7WG2* (basta resistant),[42] *pLOK180_pFR7m34GW* (gift from Lothar Kalmbach, Cambridge, similar backbone as pB7m34GW but with the basta resistance replaced by a FASTRED cassette for selection of transgenic seeds via red fluorescence) or pMDC32[43] (hygromycin resistant) destination vectors.

The following Gateway LR reactions were set-up to generate the corresponding destination vectors (the name of the corresponding transgenic line is highlighted in bold):

2x35Sprom::MAKR2-2xmCHERRY/pH7m34GW (**MAKR2-Ox1**) was obtained by recombining 2x35Sprom/pDONR4-P1R, MAKR2gnoSTOP/pDONR221, 2xmCHERRY-4xMyc/pDONR2R-P3, and pH7m34GW.

2x35Sprom::MAKR2-mCITRINE/pB7m34GW (**MAKR2-Ox2**) was obtained by recombining 2x35Sprom/pDONR4-P1R, MAKR2gnoSTOP/pDONR221, mCITRINE/pDONR2R-P3, and pB7m34GW.

2x35Sprom::amiMAKR2/pMDC32 (**amiMAKR2.1** and **amiMAKR2.3**) 2x35Sprom::amiMAKR2/pB7WG2 (**amiMAKR2.2**) were obtained by recombining amiMAKR2/pDONR221, with pMDC32 or pB7WG2, respectively.

MAKR2prom::MAKR2-2xmCHERRY/pH7m34GW (**MAKR2::MAKR2-2Ch**) was obtained by recombining MAKR2prom/pDONR4-P1R, MAKR2gnoSTOP/pDONR221, 2xmCHERRY-4xMyc/pDONR2R-P3, and pH7m34GW.

MAKR2prom::MAKR2-tdYFP/pB7m34GW (**MAKR2::MAKR2-tdYFP**) was obtained by recombining MAKR2prom/pDONR4-P1R, MAKR2gnoSTOP/pDONR221, tdYFP/pDONR2R-P3, and pB7m34GW.

MAKR2prom::MAKR2-GUS/pK7m34GW (**MAKR2::MAKR2-GUS**) was obtained by recombining MAKR2prom/pDONR4-P1R, MAKR2gnoSTOP/pDONR221, GUS/pDONR2R-P3, and pK7m34GW.

MAKR2prom::VENUS^{NLS}/pB7m34GW (**MAKR2::VENUS^{NLS}**) was obtained by recombining MAKR2prom/pDONR4-P1R, VENUS^{NLS}/pDONR221, mock/pDONR2R-P3, and pB7m34GW.

TMK1prom::CHERRY^{NLS}/pH7m34GW (**TMK1::2Ch^{NLS}**) was obtained by recombining TMK1prom/pDONR4-P1R, mCHERRYnoSTOP/pDONR221, mCHERRY^{NLS}/pDONR2R-P3, and pH7m34GW.

UBQ10prom::TMK1-2xmCHERRY/pH7m34GW (**TMK1-Ox**) was obtained by recombining UBQ10prom/pDONR4-P1R, TMK1noSTOP/pDONR221, 2xmCHERRY-4xMyc/pDONR2R-P3, and pH7m34GW.

UBQ10prom::TMK1^{K616R}-2xmCHERRY/pH7m34GW (**TMK1^{K616R}-Ox**) was obtained by recombining UBQ10prom/pDONR4-P1R, TMK1^{K616R}noSTOP/pDONR221, 2xmCHERRY-4xMyc/pDONR2R-P3, and pH7m34GW.

2x35Sprom::MAKR2-mCITRINE^{GEGE}/pB7m34GW (**MAKR2-mCit^{GEGE}**) was obtained by recombining 2x35Sprom/pDONR4-P1R, MAKR2(CDC)/pDONR221, mCITRINE^{GEGE}/pDONR2R-P3, and pB7m34GW.

2x35Sprom::MAKR2-mCITRINE^{GEGE}/pLOK180_pFR7m34GW (**MAKR2^{11Q}-mCit**) was obtained by recombining 2x35Sprom/pDONR4-P1R, MAKR2^{11Q}(CDC)P/pDONR221, mCITRINE/pDONR2R-P3, and pLOK180_pFR7m34GW.

Cloning and characterization of makr2 crispr allele

The *makr2-1* crispr allele (Col0) was generated using the egg cell-specific promoter (*pEC1.2*) CRISPR/Cas9 system described in Wang et al.[52] Two single guide RNAs (sgRNA) were originally used in the construct: *sgRNA3 5′-CTTTCAGTCTCCTTAACTAC-3′* and *sgRNA4 5′-TCTCTTTGCCGTTACTCCG-3′* but mutations were only found in the sgRNA3 target sequence. Primers containing the sgRNA sequences and the BsaI restriction site were designed (F1 *gRNA3: ATATATGGTCTCGATTGCTTTCAGTCTCCTTAAC TACGTT*; F0 *gRNA3:TGCTTTCAGTCTCCTTAACTACGTTTTAGAGCTAGAAATAGC*; *R0 gRNA4: AACGCGGAGTAACGGCAAAGA GACAATCTCTTAGTCGACTCTAC; R2 gRNA4: ATTATTGGTCTCGAAACGCGGAGTAACGGCAAAGAGACAA*) and a fourth primer PCR was performed using the *pCBC-DT1T2* vector as a template. The PCR fragment containing the sgRNAs was introduced to the binary vector *pHEE401E* by golden gate cloning using BsaI restriction sites. Plants were transformed and T1 plants selected by hygromicin resistance. Plants carrying mutations were selected by sequencing and, homozygous mutant plants depleted of the T-DNA were counterselected in T2. The *makr2-1* mutant carries a frameshift mutation (1 nucleotide (A) insertion) at position 25 relative to the *MAKR2* start ATG. The resulting predicted short MAKR2 protein comprises 37 amino acids. The first 8 amino acids correspond to MAKR2 wt sequence, and amino acids 9 to 37 represent an aberrant amino acid sequence.

Gravitropism experiments

The horizontal gravitropic index was quantified using imageJ as indicated in Platre et al.[25] and Grabov et al.[53] Plants were grown on plates with a 45° angle. The horizontal growth index was calculated on 7-day-old seedlings using the "segment line" tool on FIJI. Briefly, to calculate the gravitropic indexes, two length are considered, L and Lx.[53] L is the total length of the roots (from base of hypocotyl to root tip), while Lx is the abscissa of the root tip (considered from the point of view of the base of hypocotyl). The horizontal growth index (HGI) corresponds to the ratio Lx/L. The experiments were performed 3 times and at least 21 plants of each genotype were quantified in every independent experiment.

Gravitropic experiments were performed either on 5 to 6-days-old seedlings grown in MS media containing 1% sucrose. In order to align the roots, one hour prior to the experiment, seedlings were transferred in a new vertical plate. An angle of 135° was applied in darkness and the same plates were scanned after 3 or 4 hours, 6 hours, 12 hours, 24 hours or 48 hours. The angle of the root bending at each time point was quantified using ImageJ.[48] The experiment was performed 3 independent times and at least 30 plants of each genotype were quantified.

Kinetics of the PIN2 dynamics during gravitropism was performed as follows: 5-days-old seedlings were transferred and aligned in new 1/2 MS Petri dishes one hour prior to the experiment. In this case, an angle of 90° was applied and fluorescent images were taken every five minutes during six hours. The angle of the root bending was quantified using ImageJ.

Quantitative RT-PCR (qPCR)

Total RNA was isolated using the Plant/Fungi RNA Purification Kit (sigma) and quantified using Nanodrop 1000 (Thermo Scientific, http://www.nanodrop.com/). 1 μg of total RNA was reverse transcribed and amplified using the SuperScript IV VILO Master Mix (Thermofisher). Transcripts levels were measured by qPCR using amplified cDNA. The relative amount of each transcript was calculated with the $2^{-\Delta CT}$ method[54] using *EF1-alpha* and *GADPH* transcripts as housekeeping for data normalization. Each experiment was performed in at least three biological replicates. The qPCR primers used are described in the table below.

Drugs and hormones treatments

Drugs and hormones treatments were performed in liquid 1/2 MS on 5 days-old-seedlings. Cycloheximide (CHX) (stock 50 mM in DMSO) and MG-132 (stock 25 mM in DMSO) treatments were carried out at 50 μM during two hours before the NAA treatment.

NAA treatments were done at 1 μM or 10 μM during 5 or 30 minutes, as indicated in the experiment. The auxin analogs 5-F-IAA (stock 10 mM in ethanol), PEO-IAA (stock 10 mM in ethanol) and benzoic acid (BA) (stock 10 mM in ethanol) were diluted in 1/2 MS at 10 μM during 30 minutes. Brassinolide (stock 10 mM in DMSO) treatments were performed at 10 μM during 30 minutes.

Each treatment was repeated in at least three independent experiments and quantifications were performed on at least 14 roots. The number of cells quantified is indicated in the corresponding graphs.

Microscopy

The experiments were performed using either the LSM710 confocal microscope using a 40X Plan-apochromatic objective (numerical aperture 1.2, oil immersion) (Zeiss) or inverted Zeiss microscope (AxioObserver Z1, http://www.zeiss.com/) equipped with a spinning disk module (CSU-W1-T3, Yokogawa, http://www.yokogawa.com/) and a ProEM+ 1024B camera (PrincetonInstrument, http://www.princetoninstruments.com/) using a 40X C-Apochromat objective (numerical aperture 1.1, water immersion).

tdYFP and mCitrine were exited at 512 nm and mCherry was excited at 550 nm. FM4-64 stainning were performed as described in Marquès-Bueno et al.[46]

MAKR2-Ox1 imaging was performed using a FV 1000 confocal microscope (Olympus, Tokyo, Japan) mCherry was excited at 550 nm and using 40X oil objective.

Microfluidic experiments were performed using a vertical ZEISS LSM700 confocal microscope with a 20x/0.8 Plan-Apochromat M27 objective.[55] In this case, tdYFP was exited at 488 nm and images were taken every minute, during six minutes. First, three images of roots in liquid MS media without hormones were acquired and afterward NAA 10μM or IAA 10 μM or benzoic acid 10μM were added and three additional pictures were taken.

The kinetics of the PIN2 dynamics was performed using a verticaly mounted Olympus MVX10 macroview fluorescence stereomicroscope,[28] setting up the automated filterwheels to allow fluorescence imaging for GFP and using 5x magnification. (http://www.olympusamerica.com). For the time lapse imaging, "Process Manage" function in Cellsens Dimension software (Olympus) was used. After finishing the acquisition, the images were saved as a video and analyzed using ImageJ software.

6-days-old seedlings of *MAKR2prom::MAKR2-GUS, DR5prom::GUS* or *MAKR2ox1-DR5prom::GUS* were fixed in cold 90% acetone on ice during 20 minutes, then washed three times with 50 mM NaHPO4 buffer (pH 7.2) and stained over night with 2 mM X- Gluc staining buffer at 37 C in the darkness. Afterward, roots were washed with ethanol series from 30%, 50%, 70% 20 min each step and then finally 100%. Finally, the roots were mounted with chloralhydrate and pictures were obtained using the Leica DM6 (Leica, Germany) epifluorescence vertical microcroscope equiped with a Normansky optics and using either 20x or 40x magnification. Quantifications were done using region-of-interests drawn in the upper and lower part of the root tip and GUS/GFP intensity were quantified using the mean intensity tool from ImageJ.

In the case of DR5prom::GUS, the analyses were made on 60 roots and three independent experiments (20 roots per replicate), and 42 roots and three independent experiments (14 roots per replicate) for DR5prom::GFP.

In vitro Halo-pull down

The cytoplasmic domains of TMK1 and HSL1 cloned into pDONR221 was recombined by LR reaction into pTNT-HA-GW.[44] The HSL1^{cyt} clone was ordered to ABRC.[47] mCITRINEwSTOP/pDONR221 and MAKR2wSTOP/pDONR221 were recombined by LR reaction into pIX:Halo:ccdb.[45] The TnT Coupled Wheat Germ Extract System from Promega were used for cell free expression. 4 μg of both plasmids were added to the reaction mix following the commercial protocol. The reactions were incubated at 25°C for 2 hours. The equivalent of 10 μl of dry Halo-tag magnetic beads from Promega were added and the samples were incubated at room temperature for 30 minutes on an orbital shaker. The supernatant was discarded and the beads were incubated for 3 minutes in PBS + 0,05% Tween20. The solution was discarded. This wash was repeated 3 times. The beads were then resuspended in SDS-PAGE loading buffer, separated by SDS-PAGE and subjected to immunoblotting using anti-Flag antibody (clone M2, F1804, Sigma) and Anti-HA antibody (12CA5, Sigma-Aldrich).

Co-expression, purification and mass-spectrometry

To generate the backbone plasmid for co-expression in bacteria, *pET28a+* and *pACYC-duet* were digested using Xba1 and Xho1. The MCS cassette of *pACYC-duet* was inserted into *pET28a+* leading to the generation of *pET28-duet*. *MAKR2* and *TMK1* were amplified by PCR from Col-0 cDNA. The tags and restriction sites were added by PCR. *MAKR2* PCR fragment was cloned into *pET28-duet* between Nde1 and Xho1 and *TMK1* PCR fragment between Nhe1 and Not1. Mutation to generate a dead kinase version of TMK1 was done by PCR. The resulting plasmids were transformed in *Escherichia coli* BL21 (DE3) Rosetta 2 from Novagen that were spread on LB + 100 μg/μL kanamycin plates. Several colonies were picked and transferred into 100ml of 2xTY + 100 μg/μL kanamycin and incubated overnight at 37C under 180rpm shaking. The day after, a 250ml culture of 2xTY + 100 μg/μL kanamycin was inoculated with the preculture to reach a 0.1 OD_{600} and incubated at 37°C under 180 rpm shaking until OD_{600} 0.6. Then 0.5 mM IPTG was added and the samples were incubated for 75 minutes at 37°C. The cultures were pelleted through a 5000 rcf centrifuge for 20 minutes. The pellets were resuspended in 15 mL of resuspension buffer (20 mM Tris pH8, NaCl 150mM and 20mM imidazole). The resuspended pellets were flash freezed and stored at −20°C. The pellets were unfrozen, 5 mM beta-mercaptoehanol was added, then sonicated and centrifuged at 15000 rcf for 40 minutes at 4°C. The soluble fraction was applied on 0.5 mL of IMAC-nickel resin. The resin was washed with 10 column volumes of resuspension buffer, then 5 column volumes of resuspension buffer + 40 mM imidazole. The proteins were eluted within 2 column volumes of Tris 20mM pH8, NaCl 150mM and 400 mM imidazole. The samples were analyzed by mass spectrometry (ESI/MS/MS) by PSF platform of SFR Biosciences (UMS3444/CNRS, US8/Inserm, ENS de Lyon, UCBL).

Co-immunoprecipitation Assay

Co-immunoprecipitation assays were performed in the *Arabidopsis* suspension culture (PSB-L) protoplast system. Protoplast were isolated and transfected or co-transfected with 15 μg of plasmid DNA (*UBQ10::TMK1-3HA, UBQ10::TMK1$^{\Delta kinase}$-3HA, 2x35S::MAKR2-mCitrine*) according to the method described previously.[56] After transfection, protoplasts were incubated for 16 hours in the dark to allow gene expression, treated with either DMSO or 1μM IAA dissolved in DMSO for 1 hour, collected and frozen in liquid nitrogen. For coimmunoprecipitation (Co-IP) assay, $5x10^5$ transfected protoplasts were lysed with 50 μl of extraction buffer (50 mM Tris HCl pH 7.5, 150 mM NaCl, 10 mM $MgCl_2$, 1% (w/v) Triton X-100, 5 mM DTT, PhosSTOP phosphatase inhibitor cocktail (Roche), 1x EDTA free-Complete Protease Inhibitor Cocktail (Roche). After vortexing vigorously for 30 s, the samples were centrifuged at 14,000 × g for 10 min at 4°C and supernatants were collected. Prior to the Co-IP assay, 25 μl of collected supernatants were analyzed by immunoblot assay to determine Co-IP input. To perform the Co-IP assay, 75 μl of extraction buffer without Triton X-100 were added to remaining 25 μl of supernatants, followed by incubation with GFP-Trap Magnetic Agarose (ChromoTek) for 1 hour at 4°C, three washing steps with washing buffer (50 mM Tris HCl pH 7.5, 200 mM NaCl, 1 mM EDTA, 0.1%Triton X-100), once with 50 mM Tris·HCl pH 7.5 and elution step with 2x-SDS-sample buffer for 10 min at 95°C. Samples were separated by SDS-PAGE and subjected to immunoblotting using anti-GFP-HRP antibody (Miltenyi Biotec) and Anti-HA-Peroxidase, High Affinity (3F10) (Roche).

To generate *UBQ10::TMK1-3HA, UBQ10::TMK1$^{\Delta kinase}$-3HA* constructs, the *TMK1full length/ TMK1$^{\Delta kinase}$* (amino acid 1-587) cDNA without stop codon were amplified by RT-PCR from WT total RNA *using TMK1-FL-B1-F* and *TMK1-FL-B2-R/TMK1-ΔKD-B2-R* primers, Key Resources Table), respectively, and inserted into the pDONR221 vector by BP recombination reaction. Next, *pDONR P4-P1R-UBQ10prom, pDONR P2R-P3-3xHA* and *pDONR221-gTMK1/gTMK1$^{\Delta kinase}$*, were cloned into *pB7m34GW* vector, respectively, by MultiSite Gateway LR recombination reaction.

Image Quantification

The quantification of PIN2-GFP fluorescence during gravitropism was performed as followed. Using ImageJ software (https://imagej.nih.gov/ij/), we draw a line (1px in width and 200px in length) on both sides of the root (upper and lower side) at each time point and used the mean intensity fluorescence tool from ImageJ for quantification. Afterward, the ratio between t/t0 (t0 being the initial fluorescence intensity at the beginning of the experiment) was applied to be able to follow the difference of fluorescence. Each graph in Figure 2 were made from three independent roots.

Quantifications of MAKR2 translocation from the PM to the cytoplasm was carried as described in Simon et al.[17] Briefly, we scored, in a double-blind set-up, the number of cells in which MAKR2-tdYFP or MAKR2-mCit was associated or not with the plasma membrane. A cell was counted as positive for plasma membrane labeling when the cell outline was at least twice as fluorescent as the cytosol. All the results are expressed as a percentage of the number of cells with MAKR2 at the plasma membrane over the total number of cells counted. The number of cells (n) counted in each case is indicated on the corresponding figure. Each experiment was repeated three time and at least 14 independent roots were counted.

For the microfluidic experiments, the quantification was performed by drawing a line at the plasma membrane and in the cytoplasm and quantifying the mean average intensity of both (ImageJ). With these values, the ratio of the fluorescence between the plasma membrane and the cytoplasm was applied and represented in the graphs.

QUANTIFICATION AND STATISTICAL ANALYSIS

Phenotyping

For the horizontal gravitropic index and the kinetics of the gravitropic response, a linear model was fitted on measurements from wild-type plants and the different mutants using lm() function from stats package available in R software (https://www.r-project.org/). This model estimates a weight for each variable (wild-type and mutant plants) and the associated probability that such weight is different from zero based on a t test. The probability derived from the t test is the p value in this comparison and significant differences were considered when the p value was lower than 0.05.

Cell biology experiments

For translocation of MAKR2 from the plasma membrane to the cytoplasm a pairwise comparison between mock plants and plants subjected to different treatments was performed using a t test with Welch correction to account for unequal variances using R software (https://www.r-project.org/). The probability derived from the t test is the p value in this comparison and significant differences were considered when the p value was lower than 0.01.

All the graphs were drawn using excel software (Microsoft, https://products.office.com/) except for the boxplots which were drawn either with excel or the R software (https://www.r-project.org/).

Current Biology, Volume *31*

Supplemental Information

Auxin-Regulated Reversible Inhibition of TMK1 Signaling by MAKR2 Modulates the Dynamics of Root Gravitropism

Maria Mar Marquès-Bueno, Laia Armengot, Lise C. Noack, Joseph Bareille, Lesia Rodriguez, Matthieu Pierre Platre, Vincent Bayle, Mengying Liu, Davy Opdenacker, Steffen Vanneste, Barbara K. Möller, Zachary L. Nimchuk, Tom Beeckman, Ana I. Caño-Delgado, Jiří Friml, and Yvon Jaillais

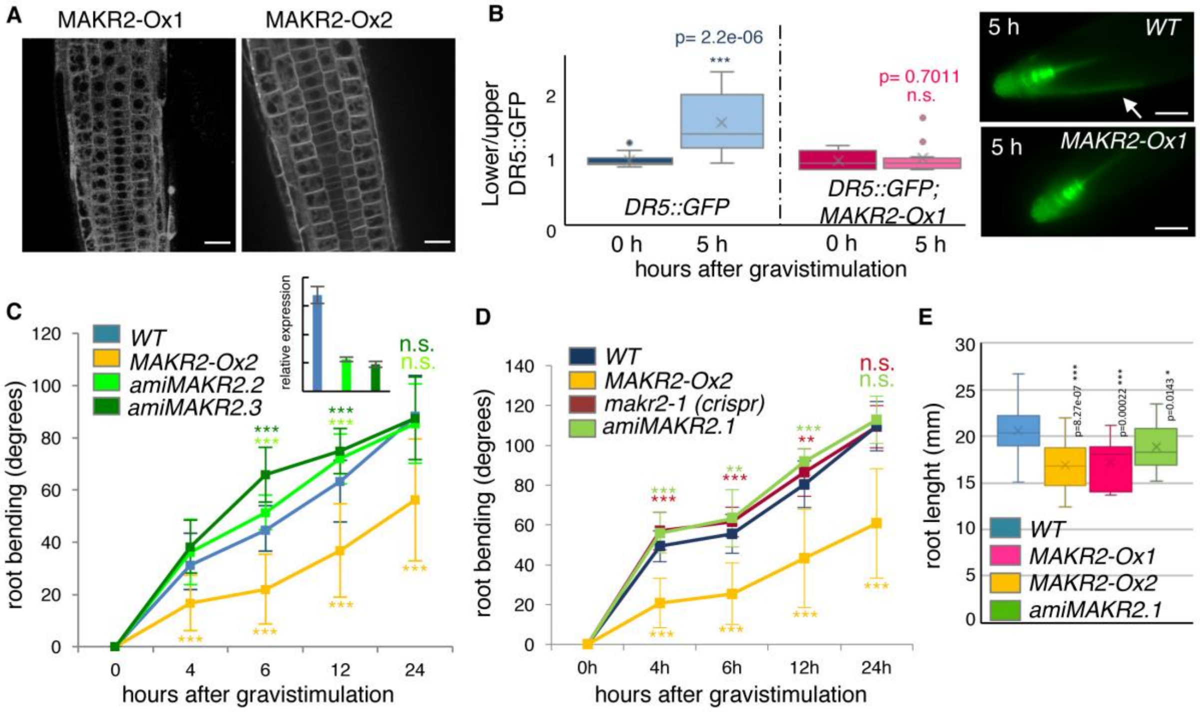

F

p-values related to Figure 1D

	6h	12h	24h	48h
MAKR2-Ox2	3.92e-07 ***	< 2e-16 ***	9.63e-10 ***	< 2e-16 ***
MAKR2-Ox1	9.93e-05 ***	7.38e-11 ***	0.00321 **	7.72e-09 ***
amiMAKR2.1	0.00184 **	0.00147 **	0.00848 **	0.983 n.s.

P-values related to Figure S1C

	4h	6h	12h	24h
MAKR2-Ox2	2.71e-10 ***	< 2.2e-16 ***	< 2.2e-16 ***	3.95E-15 ***
amiMAKR2.2	0.01531 *	2.03e-05 ***	1.69e-06 ***	0.7413 n.s.
amiMAKR2.3	0.0007991 ***	3.30e-05 ***	5.14e-11 ***	0.7967 n.s.

P-values related to Figure S1D

	4h	6h	12h	24h
MAKR2-Ox2	<2e-16 ***	< 2.2e-16 ***	< 2.2e-16 ***	<2.2e-16 ***
amiMAKR2.1	0.000245 ***	0.00124 **	4.57e-05 ***	0.157 n.s.
makr2-crispr	1.01e-05 ***	0.000374 ***	0.00794 **	0.902 n.s.

p-values related to Figure 2C

	6h	12h	24h
MAKR2-Ox1	1.37e-07 ***	6.42e-08 ***	2e-16 ***
ROP6-Ox	0.77 n.s.	0.00261 **	0.00379 **
MAKR2-Ox1;ROP6-Ox	0,119 n.s.	0,227 n.s.	0,0648 n.s.

Figure S1. Characterization and phenotyping of *MAKR2* gain- and loss-function mutants. Related to Figure 1 and 2. A) confocal picture showing the expression and localization of MAKR2-2Ch (*MAKR2-Ox1* line) and MAKR2-mCit (*MAKR2-Ox2* line). Scale bars: 30 µm. **B)** Quantification of DR5::GFP asymmetry (ratio of lower/upper) before and after 5 hours gravistimulation at a 135° angle and related representative fluorescent images after 5 hours of gravistimulation. The white arrow indicate the accumulation of the DR5rev::GFP signal on the lower part (gravistimulated side) of the root in the wild type. Scale bars: 50 µm. **C)** Kinetics of root gravitropic bending after reorienting seedlings of the genotypes indicated in the top left corner at a 135° angle. See S1F for a statistical comparison of each mutant genotype with the wild type at each time point. The inset represents qRT-PCR analyses of *MAKR2* expression in wild-type and two additional independent *amiMAKR2* lines (*amiMAKR2.2* light green and *amiMAKR2.3* dark green). **D)** Kinetics of root gravitropic bending after reorienting seedlings of the genotypes indicated in the top left corner at a 135° angle. See figure S1F for a statistical comparison of each mutant genotype with the wild type at each time point. **E)** Quantification of the primary root length of the indicated genotypes. All quantitative measurements presented in different graphs have been treated as independent experiments (e.g. different wild-type). **F)** Tables showing the p-value, corresponding to the statistical comparison of each mutant genotype with the wild type at each time point of the gravitropic kinetics presented in Figure 1D, Figure S1C, S1D and Figure 2C. Signif. codes: 'n.s.' p>0.05, '*' 0.05>p>0.01, '**' 0.01>p>0.001, '***' 0.001>p.

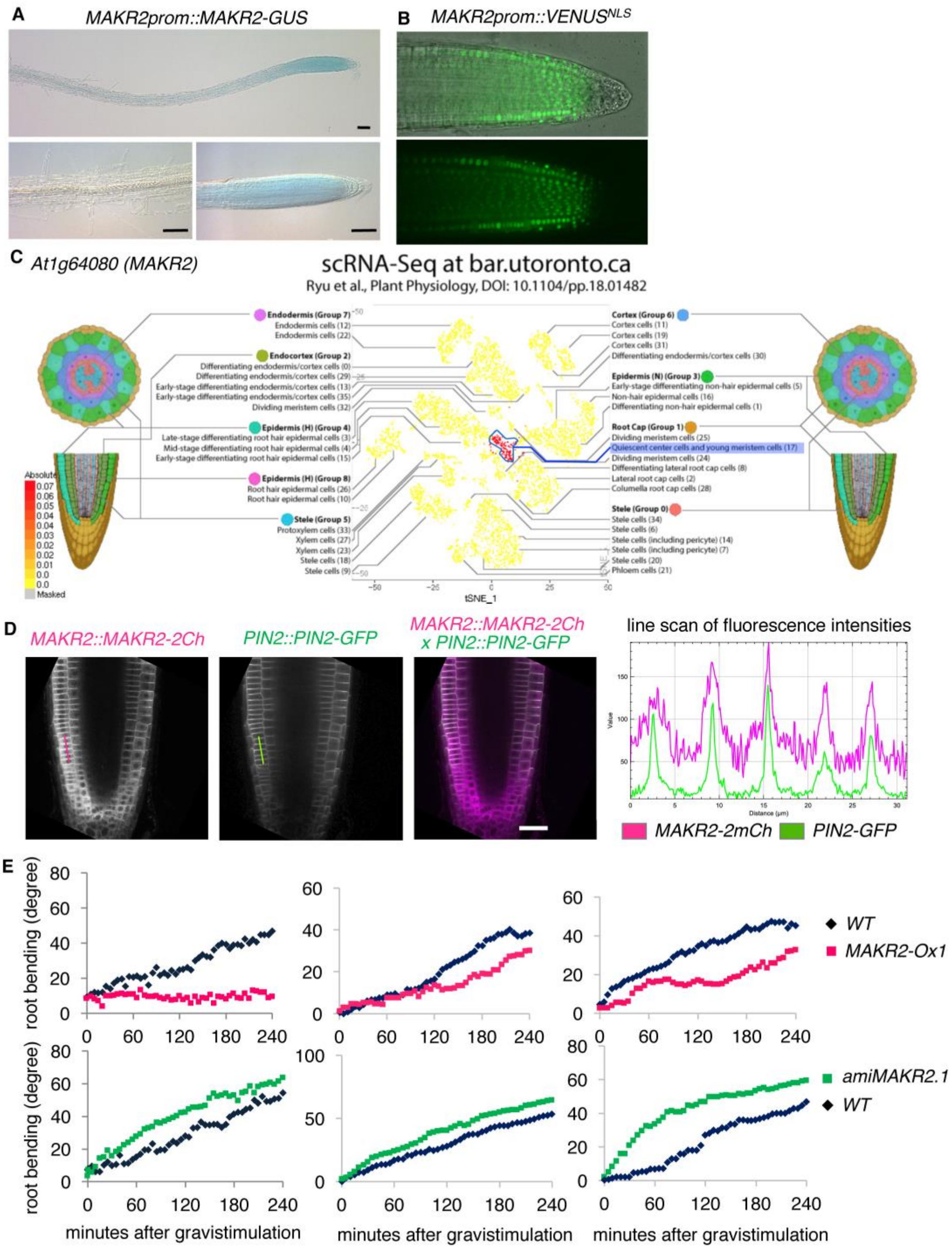

Figure S2. Analysis of *MAKR2* expression pattern in the root. Related to Figure 2. Microscopy pictures of roots showing the expression pattern of **(A)** *MAKR2prom::MAKR2-GUS*, scale bars: 100 µm and **(B)** *MAKR2prom::VENUS*^{NLS}, scale bar: 50 µm. **C)** Schematic representation of the results from single cell RNAseq for *MAKR2* expression in the root, showing that cells expressing *MAKR2* cluster together in a group of cells corresponding to quiescent center and young meristem cells. The graphic representation is from the Bio-Analytical Resource for Plant Biology (BAR, Toronto, https://bar.utoronto.ca/efp/cgi-bin/efpWeb.cgi?dataSource=Single_Cell) and the data are from [1]. **D)** Left: single channel (grey scale) of *MAKR2prom::MAKR2-2Ch* and *PIN2prom::PIN2-GFP* together with the corresponding overlay (MAKR2-2Ch in magenta, PIN2-GFP in green and colocalization in white). Right: line scan analysis of the fluorescent intensities in each channel. Note the overlapping pics, likely corresponding to the accumulation of both proteins at the plasma membrane. **E)** High resolution kinetics of the gravitropic response of *PIN2prom::PIN2-GFP*, *PIN2prom::PIN2-GFPxMAKR2-Ox1* and *PIN2prom::PIN2-GFP,xamiMAKR2.1* lines. Each graph shows the response of individual roots. See also Video S1 and S2.

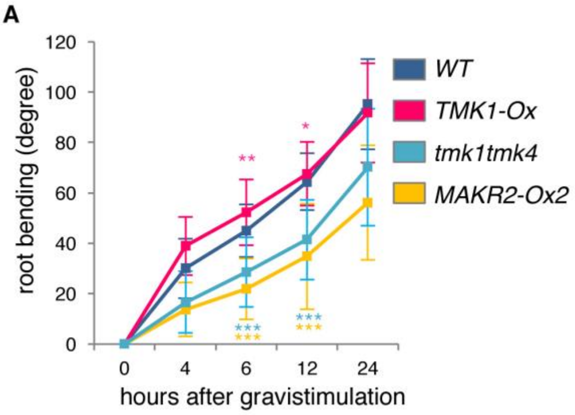

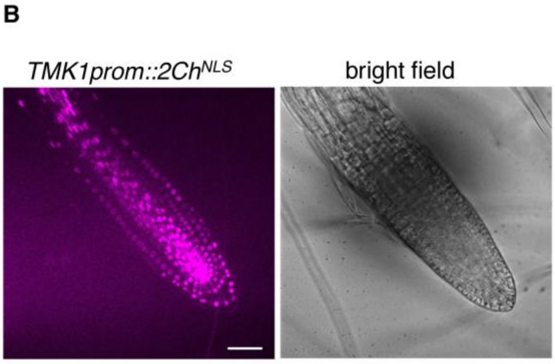

C

p-values related to Figure S3A

	6h	12h	24h
MAKR2-Ox2	1.03e-08 ***	5.82e-11 ***	1.93e-10 ***
TMK1ox	0.00824 **	0.0134 *	0,247
tmk1tmk4	3.26e-05 ***	5.05e-07 ***	8.51e-10 ***

p-values related to Figure 3A

	6h	12h	24h
MAKR2-Ox2	1.03e-08 ***	5.82e-11 ***	1.93e-10 ***
TMK1ox	0.00824 **	0.0134 *	0,247
TMK1ox;MAKR2-Ox2	0,729	0,556	0.0488 *

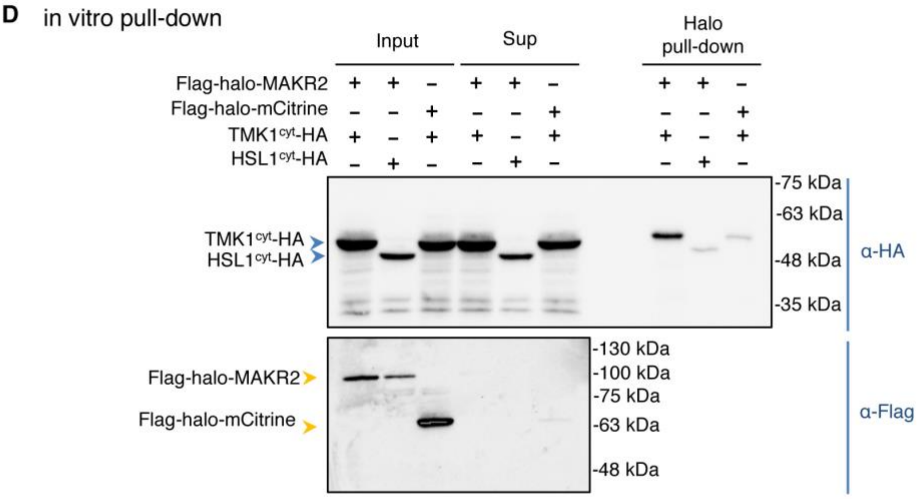

Figure S3. Quantitative analysis of the gravitropic phenotypes of *tmk1;tmk4* double mutant, expression pattern of *TMK1* and Halo-pull down with additional controls. Related to Figure 3. A) Kinetics of root gravitropic bending after reorienting seedlings of the genotypes indicated in the top left corner at a 135° angle. **B)** Confocal picture (left) and corresponding bright field image (right) showing the expression profile of *TMK1* in the *TMK1prom:2Ch*^{NLS} transcriptional reporter line. Scale bar: 50 µm. **C)** Table showing the p-value at each time point of the gravitropic kinetics presented in Figure S3A and Figure 3A. Data for WT, *MAKR2-Ox2* and *TMK1-Ox* are the same in graphs S3A and 3A. Signif. codes: 'n.s.' p>0.05, '*' 0.05>p>0.01, '**' 0.01>p>0.001, '***' 0.001>p. **D)** Pull-down assay using *in vitro* transcribed/translated proteins and Halo-tag purification. Co-purified proteins were visualized using an anti-HA antibody (labelled as Halo pull-down). The inputs (labelled Inputs) and supernatant (labelled Sup) were tested to show the relative amounts of Halo- and HA-tagged proteins and the binding efficiency to HaloLink magnetic beads (as described in [2]). TMK1^{cyt} and HSL1^{cyt} correspond to the isolated cytoplasmic domains and TMK1 and HSL1, respectively.

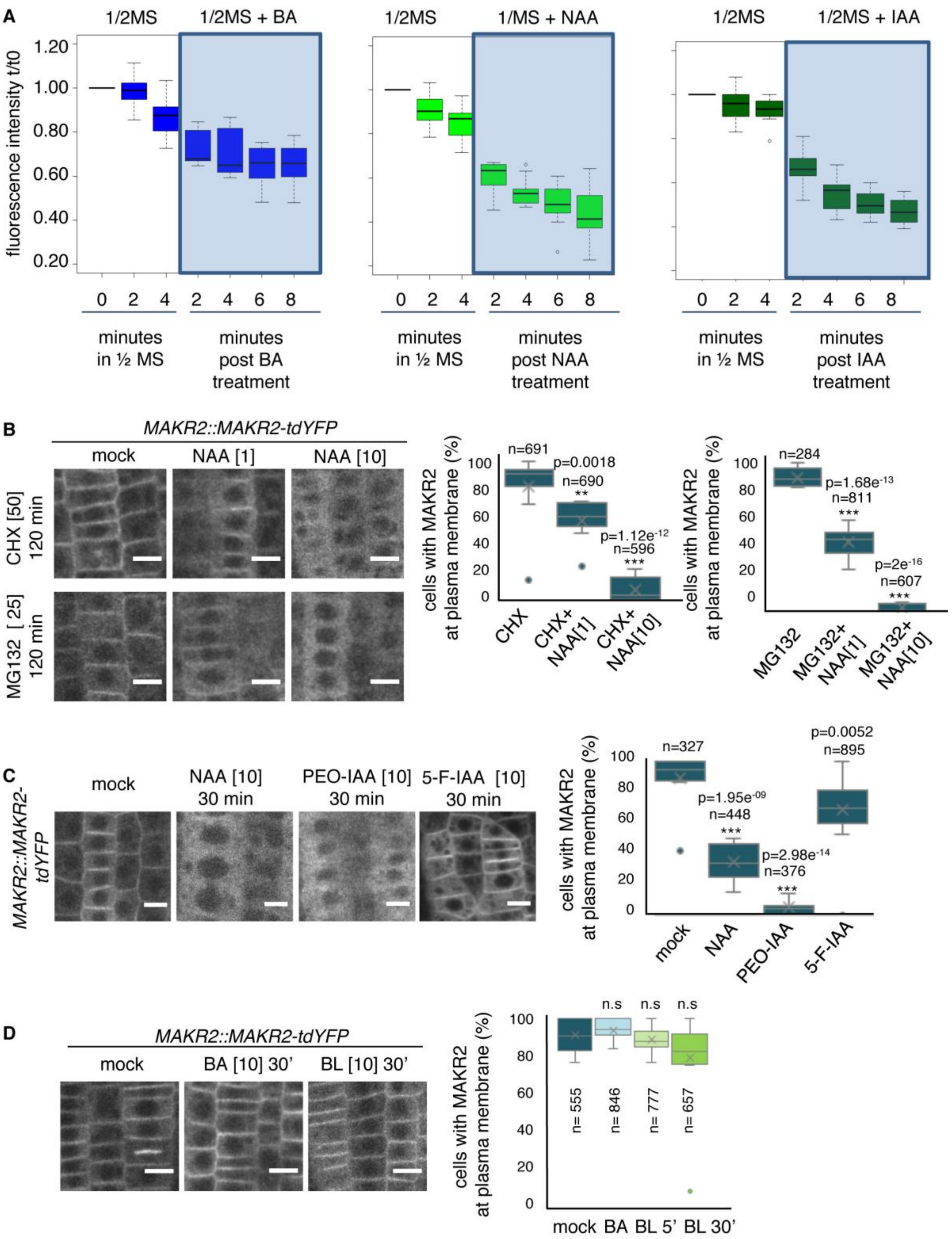

Figure S4. Time lapse analysis of MAKR2 membrane release upon auxin treatment and quantification of CHX, MG132, PEO-IAA, 5-F-IAA, BA, and BL effects on MAKR2-tdYFP localization. Related to Figure 4. A) Quantification of the MAKR2 localization at the plasma membrane and the cytosol after the application of NAA, IAA or benzoic acid (BA) treatment. Y-axis represents the ratio between the relative fluorescence at the plasma membrane at time t over the fluorescence at the plasma membrane at time t0 and the X-axis represents the time in minutes. The first four minutes shows the localization before the treatment and after either 10 µM NAA, 10 µM IAA or 10 µM benzoic acid was applied. Note that because we used a vertical confocal microscope with 20X objective with a low NA and a 488nm laser (rather than the high NA 40X objective and

515nm laser used in the rest of the experiments), we had to use a higher laser intensity accounting for increased background (i.e. fluorescence in intracellular bodies) and faster photobleaching. Comparison between the quantification of the NAA/IAA and the BA treatments allowed us to evaluate the extent of photobleaching vs auxin effect. See also Video S3. **B)** Confocal pictures and related quantifications of the *MAKR2prom::MAKR2-tdYFP* line pretreated for 120 min with the protein synthesis inhibitor cycloheximide (CHX at 50 µM) or the proteasome inhibitor MG132 (25 µM) and followed by a 30 min NAA/CHX or NAA/MG132 co-treatment at the indicated concentration of NAA. Scale bars: 10 µm. **C)** Confocal pictures of the *MAKR2prom::MAKR2-tdYFP* line following a 30 min treatment with EtOH (mock), NAA (10 µM), PEO-IAA (10 µM) and 5-F-IAA (10 µM) and related quantifications of the percentage of cells with MAKR2-tdYFP signal at the plasma membrane over the total number of cells. Scale bars: 10µm. **D)** Confocal pictures and related quantifications of *MAKR2prom::MAKR2-tdYFP* lines following a 30min treatment with either benzoic acid (BA) or brassinolide (BL). Scale bars: 10µm. n in each graph indicates the total number of cells counted.

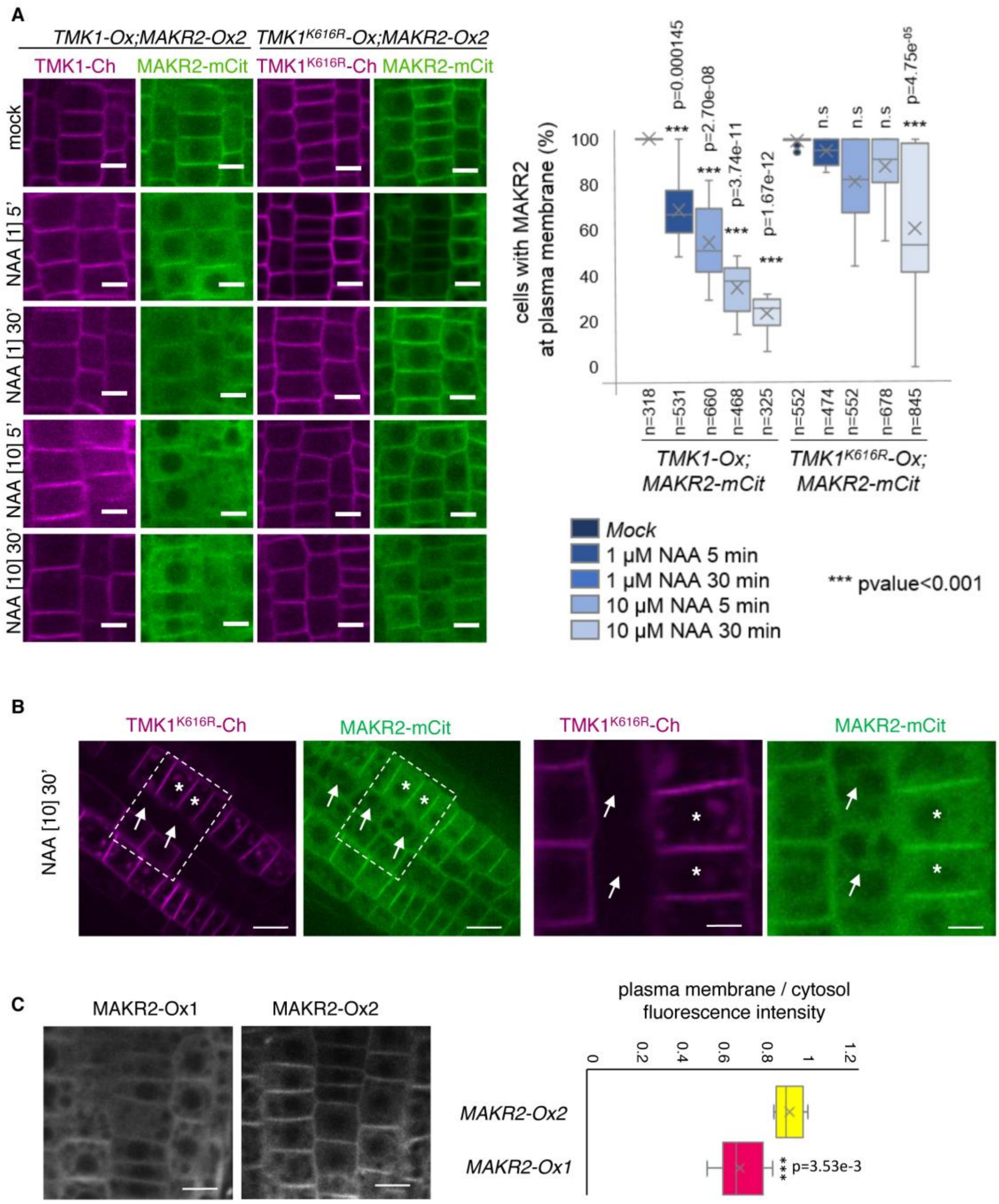

Figure S5. Quantification of TMK1-Ox and TMK1^{K616R}-Ox effect on MAKR2-mCit localization. related to Figure 4. A) Confocal pictures of roots coexpressing *UBQ10prom::TMK1-2xmCherry* (*TMK1-Ox*, left) and *2x35Sprom::MAKR2-mCitrine* (*MAKR2-Ox2*, right) (left) and *UBQ10prom::TMK1^{K616R}-2xmCherry* (*TMK1^{K616R}-Ox*, kinase dead, left) and *2x35Sprom::MAKR2-mCitrine* (*MAKR2-Ox2*, right) (right) according to the NAA treatment indicated on the left and related quantification. n indicates the number of cells counted. Note that the MAKR2-mCit picture for mock and NAA 10 µM 30 min are the same than in Figure 4C. Scale bars: 10 µm. **B)** Confocal pictures of roots coexpressing *2x35Sprom::MAKR2-mCitrine* (*MAKR2-mCit*) and *UBQ10prom::TMK1^{K616R}-2xmCherry* (*TMK1^{K616R}-Ox*, kinase dead) showing the mosaic expression of TMK1^{K616R}. The white asterisks indicate cells which express TMK1^{K616R}, and in which MAKR2-mCit does not relocalize to the cytosol following a 30-min treatment with 10 µM NAA. By contrast, the white arrows indicate cells which does not express TMK1^{K616R}, and in which MAKR2-mCit is cytosolic after a 30-min treatment with 10 µM NAA. Scale bars: 20 µm (left), 5 µm (right). **C)** Quantification of the fluorescence ratio of MAKR2 plasma membrane over cytosolic signal in *MAKR2-Ox1* and *MAKR2-Ox2* overexpression lines. Note that the relative amount of MAKR2 at the plasma membrane correlates with the level of expression (see Figure 1A) and with the respective phenotypes of these lines (see Figure 1B and 1D).

Supplemental Reference

S1. Ryu, K.H., Huang, L., Kang, H.M., and Schiefelbein, J. (2019). Single-Cell RNA Sequencing Resolves Molecular Relationships Among Individual Plant Cells. Plant physiology *179*, 1444-1456.

S2. Yazaki, J., Galli, M., Kim, A.Y., and Ecker, J.R. (2018). Profiling Interactome Networks with the HaloTag-NAPPA In Situ Protein Array. Curr Protoc Plant Biol *3*, e20071.
